# Parasite and vector circadian clocks mediate efficient malaria transmission

**DOI:** 10.1038/s41564-025-01949-1

**Published:** 2025-03-31

**Authors:** Inês Bento, Brianna A. Parrington, Rushlenne Pascual, Alexander S. Goldberg, Eileen Wang, Hani Liu, Helene Borrmann, Mira Zelle, Nicholas Coburn, Joseph S. Takahashi, Joshua E. Elias, Maria M. Mota, Filipa Rijo-Ferreira

**Affiliations:** 1 Gulbenkian Institute for Molecular Medicine, Lisbon, Portugal; 2 Berkeley Public Health, Molecular and Cell Biology Department, University of California Berkeley, Berkeley, CA USA; 3 Chan Zuckerberg Biohub – San Francisco, San Francisco, CA USA; 4 Department of Neuroscience, Peter O’Donnell Jr. Brain Institute, University of Texas Southwestern Medical Center Dallas, Dallas, TX USA; 5 Howard Hughes Medical Institute, University of Texas Southwestern Medical Center Dallas, Dallas, TX USA; 6 Faculdade de Medicina da Universidade de Lisboa, Lisbon, Portugal

**Keywords:** Parasitology, Circadian rhythms, Transcriptomics

## Abstract

Malaria transmission begins when *Anopheles* mosquitos deposit saliva and *Plasmodium* parasites during a bloodmeal. As *Anopheles* mosquitos are nocturnal, we investigated whether their salivary glands are under circadian control, anticipating bloodmeals and modulating parasite biology for host encounters. Here we show that approximately half of the mosquito salivary gland transcriptome, particularly genes essential for efficient bloodmeals such as anti-blood clotting factors, exhibits circadian expression. Furthermore, measuring haemoglobin levels, we demonstrate that mosquitos prefer to feed and ingest more blood at nighttime. Notably, we show a substantial subset of the salivary-gland-resident parasite transcriptome cycling throughout the day, indicating that this stage is not transcriptionally quiescent. Among the sporozoite genes undergoing rhythmic expression are those involved in parasite motility, potentially modulating the ability to initiate infection at different times of day. Our findings suggest a circadian tripartite relationship between the vector, parasite and mammalian host that together modulates malaria transmission.

## Abstract (web summary)

Mosquito salivary glands and malaria parasites exhibit daily rhythms, which impact blood feeding and sporozoite gene expression.

## Main

Malaria, a life-threatening disease transmitted by female *Anopheles* mosquitos, continues to pose a substantial global health and economic burden^1^. Malaria transmission is dependent on a mosquito’s behaviour of seeking a bloodmeal, which occurs predominantly at night^2–8^. This circadian-driven behaviour allows infected mosquitos to deposit *Plasmodium* parasites into the skin of a mammalian host at a specific time of the day. During a bloodmeal, uninfected mosquitos can also ingest transmissible parasite forms (gametocytes) from an infected host, which, upon ingestion, undergo sexual reproduction to form oocysts in the mosquito’s midgut, giving rise to thousands of motile sporozoites. The sporozoites then travel through the mosquito’s haemolymph, until they reach the salivary glands^9,10^, where they reside for several days ready to encounter another mammalian host and establish a new infection.

While probing the skin for a bloodmeal, female mosquitos inject saliva into the host dermis. Saliva modulates host responses including platelet aggregation, coagulation, thrombin activation, vasodilation and even inflammatory response^11–13^. Multiple proteins produced in the salivary glands are secreted in the saliva, such as *Anopheles*-specific SG1 (salivary gland 1) gene family (including TRIO^14^), apyrase^15^ and anopheline anti-platelet protein (AAPP)^16^. These saliva proteins have been shown to be important for efficient bloodmeals^15,17^. Interestingly, saliva proteins can impact parasite transmission by increasing parasite load during infection^18,19^. Saliva ingested together with blood also modulates parasite development in the midgut of the mosquito^15,20^. In addition, antiserum against salivary gland extracts has also been shown to decrease *Plasmodium* infection in rodent models^21^. Parasites may modulate vector physiology during infection, as some saliva proteins are highly expressed in infected mosquitos compared with uninfected^13,22^. Together, these studies highlight how understanding salivary gland biology may promote the development of strategies for combatting this parasite.

Various mammalian tissues exhibit daily rhythmic gene expression that regulates physiological functions^23–26^. Similarly, despite a lack of tissue resolution, *Anopheles* mosquitos were shown to possess transcripts with circadian rhythmic expressions^27,28^, probably controlled by core clock genes such as *period* (*per*), *timeless* (*tim*) and *clock* (*clk*). These clock genes, along with environmental cues, coordinate *Anopheles* pheromone synthesis, swarming and mating behaviours^29^. Alongside other molecular components, these clock genes may maintain the robustness and precision of the mosquito’s internal clock, ultimately influencing its feeding time and potentially the transmission of malaria parasites. However, it is unknown whether mosquito salivary glands are under circadian control and whether these rhythms influence the daily biology of sporozoites. Considering female mosquitos’ cyclic feeding behaviour and the importance of the salivary glands during bloodmeals, we hypothesize that a subset of salivary-gland genes would be transcribed every 24 h. This circadian rhythmic expression profile would allow the anticipation of and preparation for bloodmeals. Sporozoites are exposed to complex signals within the mosquito’s salivary glands^30^, where changes throughout the day might influence not only their biological timing but also parasite transmission and, consequently, disease outcomes.

In this study, we unveiled the dynamic nature of the mosquito’s salivary gland transcriptome and its relationship with the circadian clock. Our transcriptomic analyses identified that a substantial proportion of genes involved in blood-feeding-related functions (including anti-blood clotting) exhibit a circadian expression within the salivary glands. Moreover, we found that salivary-gland sporozoites also have a daily rhythmic transcriptome, particularly concerning genes associated with parasite transmission. These findings suggest that cyclic molecular changes in both the mosquito and parasites prepare them for bloodmeals. We propose that the interaction of host, parasite and mosquito vector circadian clocks is critical for efficient *Plasmodium* transmission. Understanding the molecular mechanisms underlying this intricate interplay will allow us to gain a deeper insight into the biology of malaria transmission, holding an immense potential for engineering multifaceted approaches to reduce disease burden. These findings may extend to other infectious diseases that cause major public health burdens such as Zika and dengue that are also vector-borne and, thus, may have daily rhythms in transmission. Ultimately, this will contribute to the global effort in the combat against these devastating infectious diseases.

## Results

### Mosquito rhythms impact feeding and salivary gland biology

**Fig. 1:**
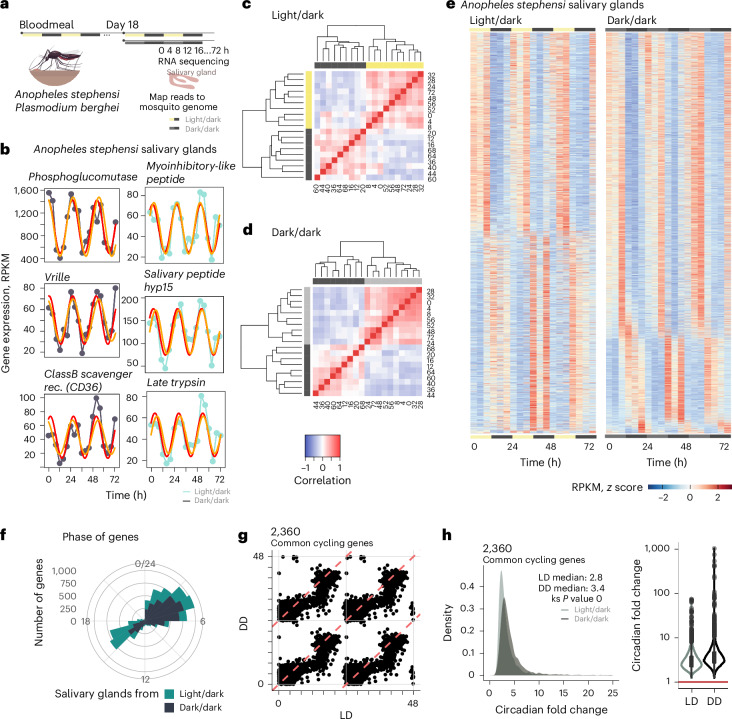
Half of the salivary gland transcriptome is rhythmic. **a**, The experimental design from two independent experiments for the sequencing of salivary glands transcripts from *A. stephensi* mosquitos infected with *P. berghei*. Infected mosquitos were maintained in cyclic conditions (12 h light/12 h dark). Eighteen days after mosquito infection, mosquitos were segregated into light/dark (LD) and constant dark (DD) conditions. Twenty-four hours later, mosquitos were dissected every 4 h over 3 days (72 h) (*n* = 32 samples for each light/dark (LD) and dark/dark (DD) condition). **b**, Representation of three genes from the top 20 cycling genes (based on significance) from each condition and the circadian-algorithm-fitted lines. **c**,**d** Hierarchical clustering and heat maps for light/dark (**c**) and dark/dark (**d**) dataset by reordering of the timepoints according to similarity in gene expression. **e**, Heat map of cycling genes from salivary glands from mosquitos in light/dark and dark/dark. Each row represents a gene. Gene expression is *z*-scored. **f**, The distribution of the peak of expression for all cycling genes in LD and DD conditions. This shows two main times of day where most of the cyclic transcription occurs. Most gene expression peaks at 4 h after lights turn on, or the beginning of daytime; and 4 h after lights off, meaning 16 h after the beginning of daytime. **g**, The phase (time of peak of expression) for each of the 2,360 common genes is largely maintained across conditions (LD and DD). The plot represents a double plot of the phase of each cycling gene in each condition. Red dashed lines denote linear correlation between the phases of 2,360 common cycling genes in each condition. Solid grey lines denote 0, 24 or 48 h timepoints on each axis. **h**, The circadian fold change in expression of the common cycling genes across conditions. The violin plot width depicts distributions of fold-change values. ks, Kolmogorov–Smirnov statistical test. Mosquito icon in panel **a** designed by Freepik. Source data

**Extended Data Fig. 1:**
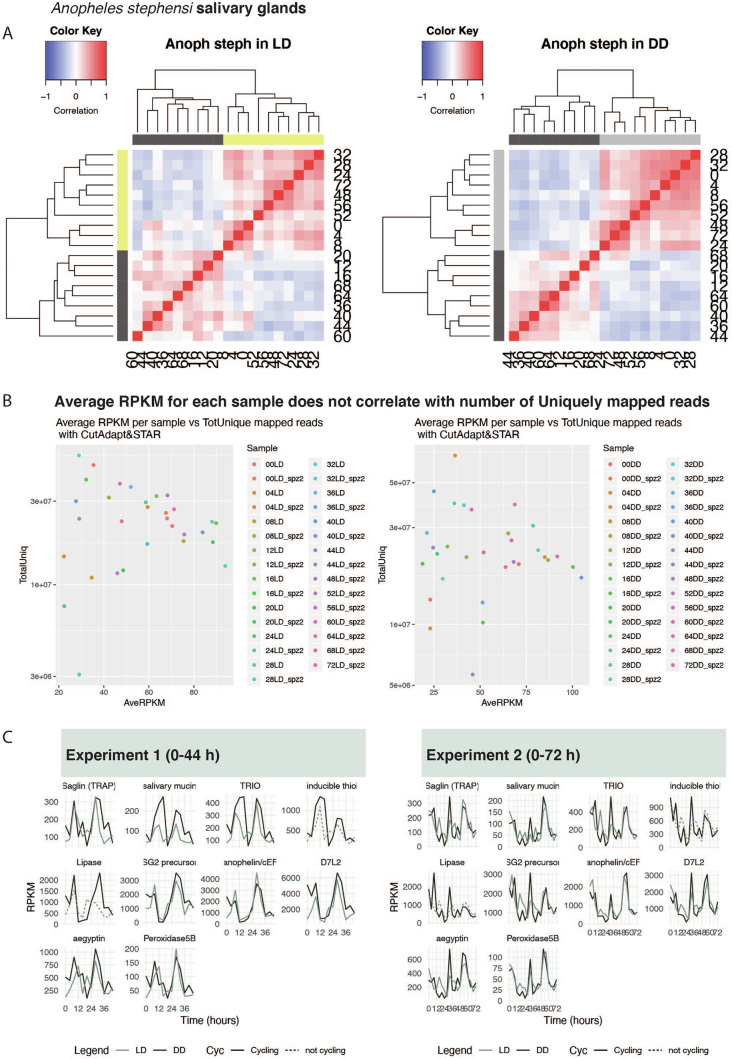
Anopheles stephensi salivary glands show transcriptional rhythms. **A**. Hierarchical clustering of mosquitos salivary gland genes in LD and DD conditions. **B**. Quality control of RPKM and total number of uniquely mapped reads. **C**. Salivary gland-specific proteins expression profile across the two experiments. Source data

**Extended Data Fig. 2:**
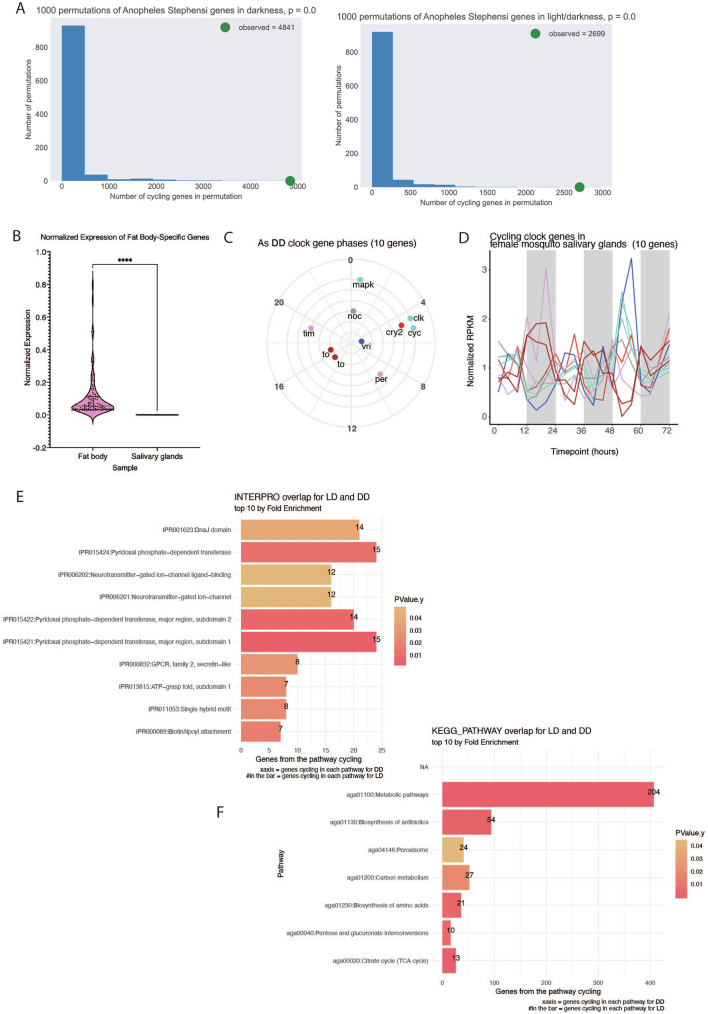
Cycling genes in mosquitos. **A**. Histograms showing that across 1,000 permutations of Anopheles stephensi genes in both light/darkness and complete darkness over 72 h, no permutation had more cycling genes than the observed sample, as determined by a combination of cycle detection algorithms. This analysis shows that an average of 70-120 genes would be defined as cycling by chance, whereas the correct time point order identified between 3000 to 5000 genes. **B**. Confirmation of salivary gland transcriptome. Normalized expression of 443 fat-body-specific genes were obtained from VectorBase (Sreenivasamurthy, Madugundu et al. 2017), where the top 100 expressed genes were plotted (pink violin plot) and compared their expression levels to our top 100 expressed fat-body-specific genes in our salivary gland transcriptome dataset in complete darkness (black horizontal line). Unpaired Welch’s t test, two-sided, ****p-value < 0.0001. **C**. Phase of known clock genes of insects. **D**. Expression profile of known clock genes of mosquitos insects. **E**. Top enriched cycling genes shared INTERPRO domains across conditions. **F**. Top enriched cycling genes shared KEGG pathways across conditions. For E/F: one-sided; used Benjamini-Hochberg (FDR) method to correct for multiple comparisons. Source data

Despite evidence that mosquitos have daily rhythms^2–8,29^, it is not known whether mosquito salivary gland biology is under circadian control nor whether this influences the daily biology of sporozoites. To investigate this, we probed the transcriptome of salivary glands from infected female *Anopheles stephensi* mosquitos kept under a 12 h light/12 h dark schedule (LD) or in constant dark (DD) for three consecutive days (Fig. 1a). We found that 27–49% of salivary-gland genes displayed a cyclic expression profile (Fig. 1b–e), five to ten times more than what has been previously reported for the mosquito’s head or body^28^. Our discovery of a high proportion of cycling genes was further supported by hierarchical clustering analysis, which clustered all daytime timepoints (light or subjective day) on the basis of genome-wide expression (Fig. 1c,d and Extended Data Fig. 1a). Most circadian clocks, including those of mosquitos, are light entrained^31,32^. A key feature of circadian clocks is that they persist with a 24-h period cycle even in the absence of environmental changes^33,34^. Genes in the salivary glands of infected mosquitos cycled independently of the mosquito light/dark schedule (LD versus DD), suggesting that they are under circadian control and that the time of day (rather than light) is the main driver of transcriptional fluctuations in mosquito salivary glands (Fig. 1e–g and Extended Data Fig. 1). By running permutation tests, we confirmed that the cycling genes identified are more than those that would be identified by chance (Extended Data Fig. 2). Together, these results reveal substantial transcriptional daily changes within mosquito salivary glands to potentially facilitate a successful bloodmeal.

Overall, salivary-gland genes reach peak expression early in the day and night (Fig. 1f,g). This ‘dual rush hour’ of circadian gene expression is well described in other organisms, such as mammals and parasites^25,35^. The 2,360 common genes that cycled in both LD and DD conditions had median oscillations of threefold amplitude and maintained their phases across conditions (Fig. 1h). Notably, some genes cycle with a circadian fold change above 100, such as arrestin-1 (ASTE007929) and guanine nucleotide-binding protein (ASTE009768).

**Fig. 2:**
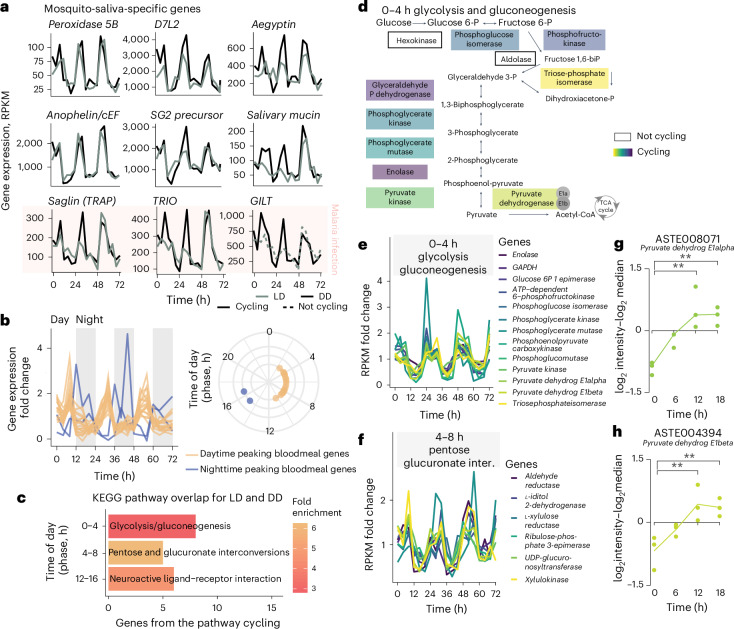
Salivary-gland-specific transcripts cycle throughout the day. **a**, The rhythmic expression profile of nine well-characterized salivary gland proteins known to have a role in bloodmeal efficiency and/or malaria transmission (*Saglin*, *TRIO* and *GILT*). **b**, The expression profile of 21 transcripts encoding for proteins associated with blood feeding, identified by being differentially expressed between female and male salivary glands^94^. Nineteen of these genes show a similar phase and profile of gene expression. Refer to Extended Data Fig. 8. Grey bars in the left plot represent nighttime or 12 h of lights off. **c**, The top significant biological functions cycling under both LD and DD conditions at specific times of day. Representative cycling genes from the pathway are from the DD condition. **d**, Representation of the glycolysis and gluconeogenesis pathway with the genes that code for the enzymes inside rectangular boxes. Only 2 out of the 11 represented genes of the glycolysis pathway do not cycle in the mosquito salivary glands throughout the day (unfilled boxes), whereas the other 9 cycle (coloured boxes). **e**,**f**, Normalized expression profile (fold-change to mean RPKM expression values across timepoints) for each gene from the DD condition belonging to the glycolysis and gluconeogenesis pathway that cycle with a maximum expression between 0 h and 4 h (**e**), and from pentose and glucuronate interconversions (inter.) between 4 h and 8 h (**f**). **g**,**h**, Normalized protein expression levels (log_2_ intensities − log_2_ median) from two salivary gland proteins across four timepoints. Normalized protein expression levels (log_2_ intensities − log_2_ median) were computed by subtracting the log_2_ median intensity value of that protein from each of its log_2_ intensity values from each replicate in each timepoint. Each point represents the normalized intensity of that protein from each replicate—a pool of five salivary glands, with three replicates per timepoint. Differential expression analysis in Extended Data Figs. 3 and 4 and Supplementary Table 1f with significance testing and fold-change calculation using MSstats. One-way analysis of variance (ANOVA), one-sided, **P = 0.0016 for ASTE008071 (**g**) and **P = 0.0096 for ASTE004394 (**h**). Source data

**Extended Data Fig. 3:**
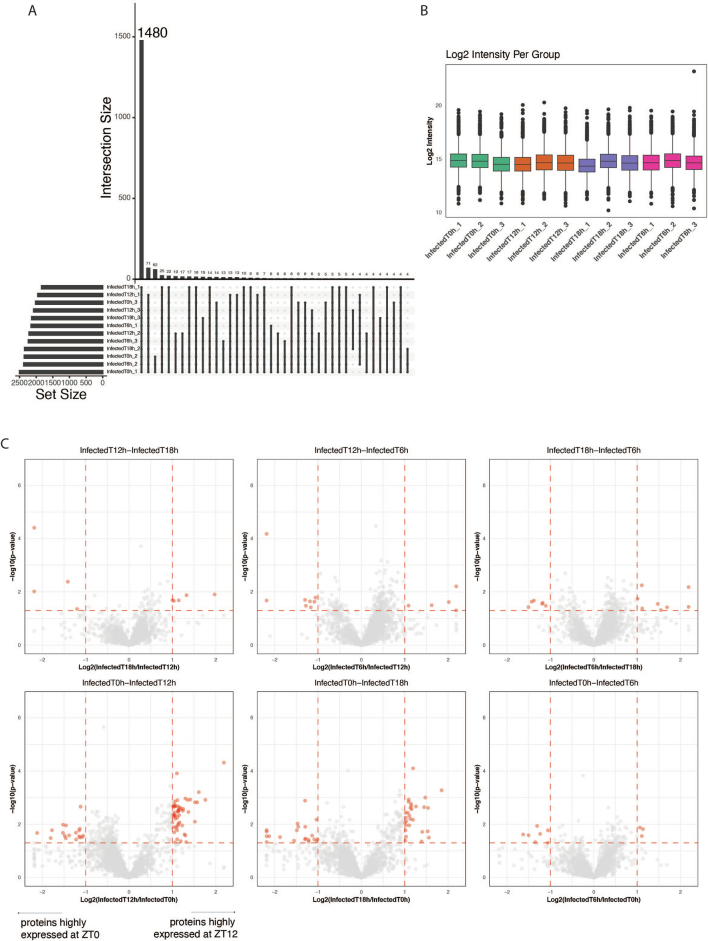
Proteomics of infected salivary glands across four times of the day. **A**. Number of proteins detected in infected A. stephensi salivary gland samples, across 3 replicates at ZT0 (Zeitgeber 0), ZT6, ZT12, and ZT18. 1,480 proteins were found to be common across all timepoints and replicates (left-most bar). Remaining bars on the plot represent the groups of proteins shared across a portion of samples but not detected across all time points and replicates. **B**. Boxplot of log2 protein intensities (protein expression) for all proteins across each of the four timepoints and its three replicates, colored by timepoint. Each replicate (each box plot of the same color) for each timepoint shows log2 intensities from all identified proteins from lysate of 5 infected salivary glands. Middle line on the boxplot indicates mean log2 intensity for all proteins detected within that replicate. For boxplot features, please refer to source data provided. **C**. Volcano plots of proteins identified from infected salivary glands (gray points) and whether they were differentially expressed (red points). Top-left panel: Proteins found to be differentially expressed (DE) at ZT12 vs ZT18. Top-middle panel: Proteins found to be DE at ZT12 vs ZT6. Top-right panel: Proteins found to be DE at ZT18 vs ZT6. Bottom-left panel: Proteins found to be DE at ZT0 vs ZT12. Bottom-middle panel: Proteins found to be DE at ZT0 vs ZT18. Bottom-right panel: Proteins found to be DE at ZT0 vs ZT6. Table S6 contains significance testing and fold-change calculation using MSstats. Red dotted line indicates cutoffs for determining DE using p-values and log2 intensities of fold change (thresholds of p-value <= 0.05, FC > = 2). Statistical analyses were performed using unpaired t-test, two-sided. Red points show DE proteins between two designated timepoints for each plot. Source data

The cycling genes in the salivary glands include the well-known circadian clock genes *Clock*, *Cycle*, *Period* and *Vrille*, as well as genes that perform other biological functions, such as *Hyp15*, which encodes an *Anopheline*-specific protein that is enriched in adult female salivary glands^36^ (Fig. 1b and Extended Data Fig. 2c–f). In fact, most of the mosquito transcripts that encode for bloodmeal-specific proteins cycled throughout the day (Fig. 2a,b). These include the vasodilator *Peroxidase 5B* (ref. ^37^), the putative anti-inflammatory *D7 long form L2* (D7L2)^38–40^, the anti-platelet aggregation *aegyptin*, anti-coagulant *anophelin/cEF* and a gene of unknown function, *Salivary Gene 2* (SG2) precursor^41^. When the expression of these genes is knocked down, the efficiency of mosquito’s bloodmeal has been shown to decline^41^. In addition, genes encoding SG1 family proteins, such as SAG (Saglin), TRIO (triple functional domain protein) and GILT (mosquito gamma-interferon-inducible lysosomal thiol reductase), that are associated with infection by malaria parasites^18–20^ also showed a cyclic profile (Fig. 2a). Saglin binds the parasite microneme TRAP (thrombospondin-related anonymous protein) protein and is relevant for midgut parasite invasion and salivary gland presence^20,42,43^. Immunization against the *Anopheles* TRIO protein can reduce the parasite burden in the host after mosquito transmission, suggesting that this protein is also important for parasitic infection^21^. GILT is a gamma-interferon-inducible thiol reductase present in the saliva of infected mosquitos that reduces *Plasmodium* sporozoite cell traversal and transmission^19^. Only female mosquitos take bloodmeals as they require specific nutrients (including proteins) from the blood to produce eggs^44^, but both males and females can survive by feeding only on plant nectar. In addition to bloodmeal-associated salivary-gland genes, we found that the glycolysis and gluconeogenesis pathways were also significantly enriched among cycling genes in LD and DD, with 9 out of 11 genes of the glycolysis pathway cycling and with maximum expression in the first 4 h of the day (Fig. 2c–f). We performed proteomics analysis comparing the levels of expressed proteins in the salivary glands proteins and found 113 differentially expressed proteins across time (out of 1,480 detected proteins across all replicates and timepoints; Fig. 2g,h and Extended Data Fig. 3a–c). These differentially expressed proteins include the two pyruvate dehydrogenases E1α and E1β of the glycolysis pathway (Fig. 2d,g,h and Supplementary Table 1f). Furthermore, we observed differences in the saliva protein content between day and night (Supplementary Table 1f). Overall, these data suggest that mosquito salivary glands have gene expression and protein abundance rhythms that correlate with the mosquitos’ feeding behaviour, in preparation for either a bloodmeal or sugar metabolism.

### Salivary-gland sporozoites exhibit rhythmic gene expression

**Fig. 3:**
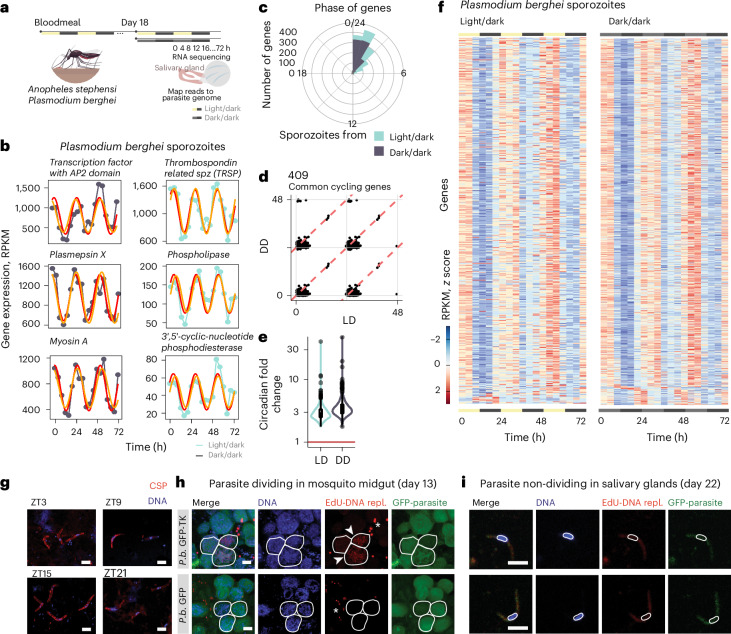
Transmission-associated genes from quiescent sporozoites have daily rhythms. **a**, The experimental design from two independent experiments for the sequencing of *P. berghei* (*P.b.*) sporozoites transcripts from *A. stephensi* mosquito salivary glands. Salivary glands were collected every 4 h over 3 days for light/dark (LD) and dark/dark (DD) conditions. **b**, Representation of six cycling genes selected from the top 20 cycling genes (based on significance) for each condition (LD and DD) and the circadian-algorithm-fitted lines. **c**, The daytime distribution of the peak expression of cycling genes showing that cycling transcription peaks between 0 h and 4 h after lights on (beginning of daytime, light phase). **d**, The phase (time of peak of expression) of the 409 common genes (that cycle in LD and DD) is maintained in both conditions. Red dashed lines denote linear correlation between the phases of 409 common genes in each condition. Solid grey lines denote 0, 24 or 48 h timepoints on each axis. **e**, Circadian fold change of the common cycling genes in both conditions. The violin plot width depicts distributions of fold-change values. **f**, Heat map of sporozoite cycling genes from mosquitos in LD and DD conditions. Each row represents a gene. The gene expression is *z*-scored. **g**, Representative immunofluorescence micrographs from purified salivary-gland sporozoites at days 22–24 after mosquito’s bloodmeal at different timepoints ZT3 (zeitgeber time, 3 h after lights on), ZT9, ZT15 and ZT21. DNA in blue and CSP in red. Scale bar, 5 µm. *n* = 20 dissected mosquitos per timepoint; two independent experiments. **h**, Representative immunofluorescence micrographs of *Anopheles* mosquito midguts with oocysts from *P. berghei*-TK-GFP and GFP (parental) line after EdU incorporation. The white circles delineate three oocysts. The arrowheads show oocysts that in the 48 h before fixation, replicated their DNA as demonstrated by the incorporation of EdU. The asterisk shows replicating mosquito midgut cells. Scale bar, 10 µm. *n* = 20 dissected mosquitos per genotype; two independent experiments. **i**, Representative immunofluorescence micrographs of salivary-gland sporozoites from *P. berghei*-TK-GFP and GFP (parental) parasite line upon feeding of mosquitos with EdU. The white line delineates a nucleus of a sporozoite. Sporozoites show no DNA replication as observed by the absence of EdU (red) at DNA (blue). Scale bar, 5 µm. *n* = 20 dissected mosquitos per genotype (repl.); two independent experiments. Mosquito icon in panel **a** designed by Freepik. Source data

The transcriptome of sporozoites has been probed extensively^45–49^. This parasite stage is often referred to as quiescent because sporozoites are thought to be cell cycle arrested and are transcriptionally similar across many days while in the salivary glands^45,46,49^. Recent single-cell RNA sequencing studies demonstrated heterogeneity within this population^45,47^. Here, by probing the rodent parasite *Plasmodium berghei* sporozoite transcriptome at a very high temporal resolution (Fig. 3a), we showed that 12–20% of sporozoite transcripts have cyclic expression (Fig. 3b,f). In addition, sporozoite genes that cycle during the day have a maximum expression early in the morning (Fig. 3c,d), with an approximate median threefold circadian change in expression and a consistent phase (that is, peak expression time) in both light conditions (Fig. 3c–e).

Once in the skin, sporozoite motility is crucial to cross the dermis and enter the blood or lymphatic systems^50^. To reach and invade their unique target, the hepatocyte, sporozoites utilize substrate-dependent gliding motility and cell traversal^51–55^. A multitude of cellular components, such as plasma-membrane proteins of the TRAP family, actin filaments, actin/myosin-based motor proteins and the microneme, a specialized organelle from the Apicomplexa phylum^55^, are critical for sporozoite motility^56^. Strikingly, myosin A and thrombospondin-related sporozoite protein (TRSP) are among the top 20 cycling sporozoite genes (Fig. 3b and Supplementary Table 1). Other genes include the transcription factor with AP2 domain and plasmepsin X, an aspartic protease important for invasion and egress during blood-stage infection but whose function in sporozoites is unknown^57,58^. Phospholipase gene expression is also rhythmic (Fig. 3b) and has been shown to be involved in the migration of sporozoites through cells^59^.

The consensus is that salivary-gland sporozoites are non-dividing parasite forms. Nonetheless, due to technical challenges, the regulation of the *Plasmodium* cell cycle has been understudied. Thus, we investigated whether the rhythmic gene expression we observed was the result of undocumented fluctuations in sporozoite numbers either due to (1) migration of sporozoites into the salivary glands at specific times of day or (2) unreported sporozoite replication within this tissue. To test whether cycling gene expression is independent of the numbers of sporozoites in the salivary glands, we normalized parasite reads by downsampling to the lowest number reads obtained in a timepoint and reanalysed for daily oscillations. We found similar results when analysing the total mapped reads or the downsampled reads (Extended Data Figs. 5 and 6a), suggesting that potential differences in daily parasite load did not account for such gene expression changes.

Because *Plasmodium* parasites have rhythmic gene expression in the mammalian blood as they divide^60–65^, we searched for evidence of cell division in salivary-gland sporozoites throughout the day. We failed to identify sporozoites with morphologies suggestive of cell division, for example, duplicated nuclei and sporozoite separation (Fig. 3g) across different circadian timepoints. To eliminate the possibility that morphological alterations due to division would be an undetectable fast event, we generated a transgenic *P. berghei* parasite line that expresses the thymidine kinase enzyme (*P. berghei*-GFP-TK). This parasite line allows the incorporation of the synthetic nucleoside analogue of thymidine, 5-ethynyl-2′-deoxyuridine (EdU), into newly synthesized DNA. We provided EdU in the food of infected *Anopheles* mosquitos and analysed its incorporation into parasites or mosquito nuclei. As expected, on day 13 post-bloodmeal, the DNA of the dividing oocyst, and midgut cells of the mosquito, showed incorporation of EdU (Fig. 3h. By contrast, no EdU was incorporated in the nucleus of the sporozoites 22 days post-bloodmeal, confirming that these are non-replicating parasite forms (Fig. 3i).

Taken together, our results show that the transcriptional daily rhythms identified in sporozoites are not a consequence of cell division but instead resemble a robust circadian rhythm. Despite being in a non-dividing stage, sporozoites are transcriptionally active parasite forms, with daily rhythms of gene expression that potentially allow parasites to anticipate timing of mosquito bloodmeal and prepare for an efficient transmission.

### Daily rhythms in sporozoite transmission

**Fig. 4:**
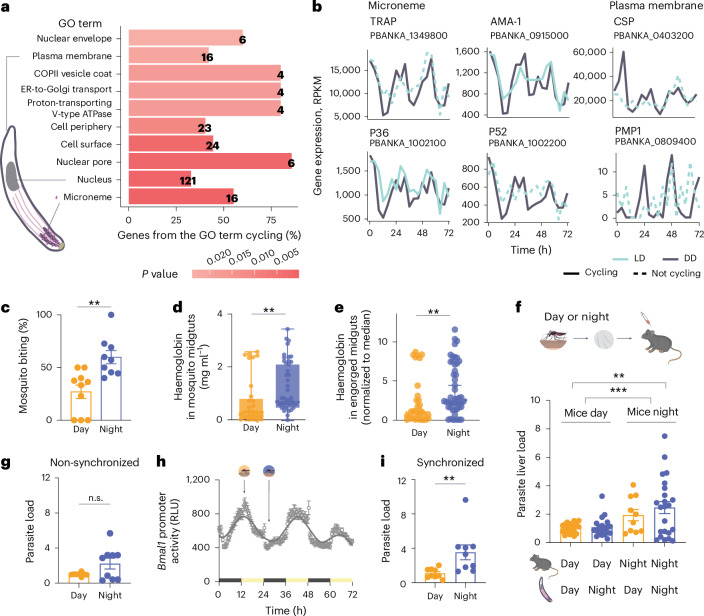
Daily rhythms in motility-associated genes and infection efficiency. **a**, The most significant biological functions with a cycling expression profile in DD and LD. Sporozoite schematics. ER, endoplasmic reticulum. **b**, Rhythmic expression profile of transcripts. **c**, The percentage of mosquitos that ingested blood within cups at ZT4 (daytime) and ZT16 (nighttime) (*n* = 9/10 cups per timepoint, *n* = 6–16 mosquitos per cup, Mann–Whitney test, **P < 0.005, two-tailed). Data are presented as mean ± s.e.m. **d**, Quantification of haemoglobin in mosquito midguts, as a proxy of blood ingested after mosquito access to a bloodmeal for 30 min (*n* = 67–68 midguts per group; four independent experiments with two replicates per timepoint; Mann–Whitney test, ***P = 0.0081, two-tailed). Data are presented as mean ± s.e.m. **e**, Quantification of haemoglobin (normalized to daytime median) in each engorged mosquito midgut, as a proxy of blood ingested, after a 30-min bloodmeal (*n* = 33–50 engorged midguts per group; four independent experiments; Mann–Whitney test, **P = 0.0082, two-tailed). For both **d** and **e**, each data point represents quantified haemoglobin from one mosquito midgut. Data are presented as median, and error bars represent 95% confidence intervals. **f**, Quantification by qPCR of parasite liver load in infected mice upon intradermal injection of sporozoites at ZT4 (daytime) and ZT16 (nighttime) in matched or mismatched schedules. The 18S expression was normalized against Hprt (*n* = 10–23 mice per group, three independent experiments, error bars represent ±s.e.m., two-tailed *t*-test, **P = 0.0024, two-way ANOVA shows significance for host timing, ***P = 0.003, Benjamini–Hochberg (FDR) method to correct for multiple comparisons). **g**, Parasite load in non-synchronized Hepa 1-6 cells upon infection with sporozoites at daytime or nighttime, assessed by qPCR normalized against Gapdh (*n* = 9 wells, error bars represent ±s.e.m. from four independent experiments; n.s., non-significant, Mann–Whitney test, two-tailed). **h**, Hepa 1-6 cells expressing luciferase under *Bmal1* promoter were cultured and entrained with temperature (*n* = 12 wells, error bars represent ±s.e.m. from one representative experiment). RLU, relative light units. **i**, Hepa 1-6 wild-type cells were entrained with temperature and infected with matching day versus night sporozoites. Parasite load was assessed by qPCR normalized against Gapdh (*n* = 9 wells, error bars represent ±s.e.m., two independent experiments, **P = 0.0056, Mann–Whitney test, two-tailed). Source data

During a bloodmeal, few parasites are injected in the mammalian host, making this an ideal stage for the use of prophylactic drugs and vaccines^66^. When we performed a functional analysis of all sporozoite cycling transcripts, the enriched Gene Ontology (GO) terms whose genes cycle both under LD and DD conditions involved microneme and plasma membrane genes (Fig. 4a), with the myosin complex among the top ten of GO terms in LD (Extended Data Fig. 7a,b and Supplementary Table 1g). Over 50% of microneme-associated genes of the parasite cycle throughout the day. This includes the apical membrane antigen 1 (AMA1) and TRAP proteins (Fig. 4b), whose mutants have impaired motility^52,56^ and whose antigenicity is being explored as vaccine candidates or monoclonal antibody treatment. Other microneme transcripts, whose protein function is essential to invade and transmigrate to the host, also cycle^67^. The circumsporozoite protein (CSP), the target of the first malaria vaccine (RTS,S)^68,69^, is essential for the initial liver invasion by binding to highly sulfated proteoglycans in the liver sinusoids^70,71^ and shows a cyclic expression (Fig. 4b). Thus, we show that sporozoites have rhythmic transcription of motility-associated genes important for invasion and infection of the mammalian host.

In the blood stage, malaria parasites have robust daily rhythms in gene expression^60–64^, and these rhythms are host independent^65^. However, there is a delay of approximately 11 h or more between the peak of mRNA transcription and protein expression^72,73^. Although knowledge of transcription–translation dynamics for malaria parasites in vivo and specifically for the sporozoite stage is unknown, we can hypothesize the existence of a complex regulatory mechanism for protein translation. In fact, highly transcribed mRNAs during the sporozoite stage, such as the parasitophorous vacuole transmembrane protein *UIS3* and *UIS4* (*UIS*, a gene upregulated in infective sporozoites)^74^, have undetectable protein content until later on in the parasite life cycle^75,76^. However, when cross-referencing a dataset of sporozoite proteins expressed in the salivary gland parasites^77^ with our cycling mRNA data, we found that 1/3 of the genes that cycle at the mRNA level are also translated into proteins in the salivary gland stage (Extended Data Figs. 6–8 and Supplementary Table 1g). These include myosin A, myosin B, myosin F, thrombospondin-related sporozoite protein, TRAP-like proteins and AMA-1. These findings suggest that some of the sporozoite mRNAs that exhibit daily rhythms are translated into protein.

Because *Anopheles* mosquitos are more likely to bite at night and many of the salivary-gland-specific genes are under circadian control (Figs. 1 and 2), this suggests that mosquitos are prepared to have a successful bloodmeal at specific times of day. Indeed, we show an increase in successful bloodmeals when mosquitos bite at nighttime (Fig. 4c and Extended Data Fig. 9a). Furthermore, we compared how much blood was ingested by each mosquito by quantifying haemoglobin levels inside the mosquito’s midgut. We observed a higher blood load in the midguts of mosquitos that fed at night (zeitgeber time ZT16; Fig. 4d and Extended Data Fig. 9b,c). To test if the time of day also influenced the amount of blood ingested by mosquitos that effectively ingested blood, we measured haemoglobin levels and found that the ones that fed during the nighttime had higher haemoglobin levels in their midguts (Fig. 4e and Extended Data Fig. 9b,d). This suggests that, even if mosquitos can feed at any time, they ingest higher blood volumes during the evening as their ability to successfully feed is also changing throughout the day. These behavioural and physiological changes across the day may have created an evolutionary pressure for sporozoites to be equipped with daily rhythms for a predictable encounter with a mammalian host at a specific time of the day. Perhaps partially due to mosquito behavioural rhythms, when infected mosquitos are allowed to feed on mice during the morning or evening, there is higher parasite load in animals at nighttime (Extended Data Fig. 9e). Next, we wanted to determine whether the time of day affects parasite transmission efficiency independently of mosquito biting. To test that, we dissected the salivary glands of infected mosquitos at the beginning of the day (ZT4) and the beginning of the night (ZT16) and infected mice with sporozoites by intradermal injection. We observed a reduced parasite load in the livers of mice when infection was initiated during the daytime (using daytime sporozoites and mice) compared with nighttime infections (using nighttime sporozoites and mice; Fig. 4f). By contrast, this increase in parasite load was abolished when the biological timing of the sporozoites and that of the mice were mismatched, regardless of whether the mismatch occurred during the day or night (Fig. 4f). To further disentangle time-of-day contributions for the host and/or parasite in transmission, we assessed the motility of sporozoites dissected from salivary glands at two different times of day in vitro, observing a trend towards increased gliding activity at night (Extended Data Fig. 9f). In addition, to eliminate the complexities of parasite traversal through different host tissues, we tested the influence of hepatocyte rhythms on parasite infection. Using a circadian reporter hepatocyte line, we examined infection loads of parasites dissected during the day or night. Parasites co-cultured with non-synchronous hepatocytes showed a trend for higher infection at night (Fig. 4g, with a mean 2.23× increased parasite load at night). When the hepatocyte population was synchronous and matched the time with sporozoites, there was a significant increase in infection at night (Fig. 4h,i, with a mean 3.76× increased parasite load at night in synchronized hepatocytes), further supporting that host circadian rhythms also contribute to the infection process. Taken together, our results suggest that the circadian regulation in both the mammalian host and the *Anopheles* mosquito vector has driven *Plasmodium* to evolve rhythms that align with both hosts, ensuring successful transmission.

## Discussion

In conclusion, we have uncovered a previously unrecognized level of mosquito–parasite interaction, revealing that sporozoites are transcriptionally active parasite forms with cyclic RNA expression, equipped to cope with host-seeking behaviour and ensure efficient infection. Our results lead us to propose that sporozoites have evolved to be rhythmic and align (or exploit) with the rhythms of vector and/or host. The daily transcriptional rhythms of sporozoites suggest that they are ‘poised’ or ‘primed’ for their crucial task of encountering and infecting the mammalian liver at a specific time, which aligns with the mosquito’s feeding time. Altogether, our study suggests that the rhythms of mosquito, parasite and host must align to maximize transmission. Notably, increased transmission happens at nighttime, coinciding with the host-seeking behaviour of the mosquito. This study characterizes the interplay of these rhythms under near-natural conditions. However, further studies are needed to dissect the contribution of each player’s physiological rhythms and clock mechanisms for the observed phenomenon. It is also likely that other layers of regulation exist in each step, for example, a central (brain-driven) and/or a peripheral regulation that shapes the mosquito’s feeding rhythm. These peripheral factors should include circadian salivary gland biology as described here, possibly regulated through a complex network including crosstalk with other organs such as the midgut. Currently, our findings highlight the importance of timing in mosquito biting and establishment of host infections, contributing to our understanding of malaria transmission biology.

We have observed almost half of the mosquito salivary glands transcriptome cycling. Such a high proportion of cycling genes, compared with previous studies^28^, could be explained by the increased sensitivity and statistical power of circadian algorithms and a longer time course at high sample frequency in our study. In addition, because previous analyses were performed from whole mosquito body parts, potential conflicting rhythms of different tissues could flatten the overall oscillations, which would lead to an underestimation of the 24-h-cycling transcripts. Alternatively, the expression of salivary-gland genes may be more rhythmic than that observed in the head and body. Here, we focused on infected *Anopheles* mosquitos; however, because many of the cycling genes encode for proteins that regulate fundamental cellular functions such as glycolysis, it is possible that these observations are extendable to uninfected mosquitos, female or male. In addition, further characterization of mosquito circadian rhythms, including salivary glands, should be done across the lifespan of the mosquito as rhythms may decline as mosquito age, similar to what was demonstrated in mammals^78^, and across multiple bloodmeals.

In the future, it will be critical to identify the key molecules responsible for signalling time-of-day information to the mosquito salivary glands and the sporozoites that infect them and explore whether these are intrinsic circadian rhythms. This knowledge could help in the exploration and identification of innovative methods to disrupt the circadian synchronization between mosquito vectors and malaria parasites, preventing efficient transmission. Nonetheless, because the rhythms of the three organisms are present in nature, the current findings describe this exciting interplay of clocks in fine tuning all rhythms. Moreover, further research is warranted by the potential impact of the use of bed nets on changing mosquito biting behaviour, new infections and parasite biology^79^. Strategies that leverage the knowledge of transmission peak time could be considered to optimize the timing of insecticide application or other vector control measures. For example, targeting insecticide spraying during periods of increased mosquito activity could maximize effectiveness. Finally, the findings of daily rhythms in salivary gland biology and disease transmission are probably applicable to other mosquito species and vector-borne diseases.

Overall, our high-temporal-resolution study provides valuable insights into the intricate interactions between mosquito vectors, malaria parasites and mammalian hosts, contributing to the advancement of intervention strategies against malaria transmission. By recognizing the active nature of sporozoites and their preparation for infection, we advance our understanding of malaria biology and open revolutionary avenues for combatting this devastating infectious disease.

## Methods

### Parasite lines

The following parasites were used in this study: GFP-expressing *P. berghei* ANKA (clone 259cl2, *Pb*-GFP); *P. berghei* ANKA-resistance-marker-free GFP-expressing line (clone 440cl1, obtained from the Leiden Malaria Research Group, http://www.pberghei.eu (ref. ^80^), and *P. berghei* ANKA-GFP line expressing the thymidine kinase (TK) from *Herpes simplex* virus (*Pb*-GFP-TK).

The *Pb*-GFP-TK parasite line was generated by double-homologous recombination targeting of the P230p locus of the GFP expressing *P. berghei* ANKA clone 440cl1. The recombination construct containing the P230p locus sequence flanking the expression cassette for the TK and the human DHFR coding sequences fused via a 2A self-cleaving peptide was kindly provided by Kathrin Witmer (Imperial College, London). Transfection was performed in blood-stage merozoites (as described by ref. ^80^). In brief, blood from a BALB/c mouse infected with *Pb*-clone 440cl1 was collected and cultured for 16 h in vitro. Mature schizonts were purified by a Nycodenz gradient and transfected with the recombination construct using the Amaxa electroporation system (Lonza). Transfected merozoites were injected into the tail vein of male BALB/c mouse (6–8 weeks of age) and selected by the administration of pyrimethamine in the mice drinking water (70 μg ml^−1^). The pyrimethamine-resistant parasite population containing the correct genomic integration of the TK/DHFR-expressing cassette was cloned by limiting the dilution and injection of one parasite per mouse (ten BALB/c male mice, 6–8 weeks of age). Genomic DNA was isolated from the blood of infected animals (NZY Blood DNA isolation kit) and the successful recombination at the modified locus was verified by PCR (NZYTech PCR mix) with the following oligonucleotide pairs: for the TK coding sequence (CAC TTG ACA GAA TGG CCT CGT; CTG CAG ATA CCG CAC CGT AT) and genomic integration (GCA AAG TGA AGT TCA AAT ATG; GGG CAT TTT CTG CTC CGG GC).

### Mosquito rearing and infections

*Anopheles stephensi* mosquitos were reared at 28 °C and 80% humidity under a 12-h/12-h light/dark cycle and fed on 10% glucose (Sigma) and 0.2% 4-aminobenzoic acid (Millipore Sigma) in MilliQ water changed daily. Female *A. stephensi* mosquitos 4–5 days old were allowed to bite two mice (BALB/c, male, 7–8 weeks of age) infected with *P. berghei* ANKA (GFP_con_, 259cl2 for 1 h). For the study of parasite DNA replication, mosquitos were infected with *P. berghei* ANKA-GFP-TK. After the infectious bloodmeal, mosquitos were kept in 12 h light/12 h dark, at a constant temperature of 20 °C. Mosquitos were fed by cotton pads soaked with 10% glucose (Sigma) and 0.2% 4-aminobenzoic acid (Millipore Sigma) in MilliQ water changed daily.

### Mice

Male C57BL/6 and BALB/c mice were purchased from Charles River or The Jackson Laboratory. Mice were maintained under specific-pathogen-free conditions and housed at 23 °C and 40–50% humidity with a 12 h light/12 h dark schedule in accordance with Portuguese law, the European Commission recommendations on housing and care of animals, the Federation of European Laboratory Animal Science Associations guidelines category B and the standards of the University of California Berkeley Institutional Care and Use Committee. Procedures involving animal experimentation received ethical approval by the Animal Welfare Body of the Instituto de Medicina Molecular (ORBEA-iMM-JLA) and were authorized by Direção Geral de Alimentação e Veterinária. In addition, all animal experiments complied with the regulatory standards of, and were approved by, the University of California, Berkeley Animal Care and Use Committee.

### DNA replication analysis

The DNA replication of parasites was assessed by analysing EdU incorporation in *P. berghei* ANKA-GFP-TK (thymidine kinase). The parental line, *P. berghei* ANKA-GFP (clone 440cl1), was also subjected to the same protocol.

Infected mosquitos were fed for 48 h or 72 h with 200 μM of EdU (Invitrogen) in the food of the mosquitos. Salivary glands from 20 infected mosquitos per timepoint per experiment (2 independent experiments) were then collected into basal DMEM (Gibco) medium on days 22–24 post-bloodmeal every 6 h for 48 h (timepoints: ZT0 (at lights on), ZT6, ZT12 (at lights off) and ZT18). Salivary glands were ground with a plastic pestle and filtered through a 40-μm Falcon cell strainer (Thermo Fisher Scientific) to release sporozoites. Sporozoites were purified using accudenz column purification method^81^. Then, 20 μl of purified sporozoites was allowed to adhere to the wells of a glass slide (Thermo Scientific ER-308B-CE24, 10 wells 6.7 mm glass) and fixed for 10 min with 4% paraformaldehyde. Sporozoites were then washed 2× in 3% bovine serum albumin (BSA) for 10 min, permeabilized for 20 min in 0.5% Triton X-100 in phosphate-buffered saline (PBS) and then washed twice for 10 min in 3% BSA. In immunofluorescence time course experiments, parasites were stained with αCSP (kindly provided by Miguel Prudêncio). For DNA replication, salivary glands from 20 infected mosquitos per genotype (2 independent experiments) were collected and purified sporozoites were subjected to the Click-iT chemistry protocol. The Click-iT chemistry reaction mix was prepared according to the manufacturer’s instructions (Invitrogen, Click-It EdU imaging kits, C10339) by addition of a fluorescent azide through a Cu(I)-catalysed reaction. Sporozoites were washed with 3% BSA in PBS, followed by a PBS wash for 10 min, incubated overnight at 4 °C with anti-UIS4 (goat, 1:500, from Sicgene) and washed three times with PBS. The secondary antibodies anti-GFP rabbit polyclonal antibody conjugated to Alexa Fluor 488 and donkey anti-goat Alexa Fluor 568 (all 1:1,000 from Life Technologies, Invitrogen) were incubated for 1 h at room temperature in 3% BSA. Cell nuclei were stained with Hoechst 33342 at 1:1,000 (Life Technologies, Invitrogen). Sporozoites were then washed 3× in PBS, and slides were mounted using Fluoromount-G (SouthernBiotech). All images were acquired on a Zeiss confocal microscope (LSM 880) with 63× amplification and processed in ImageJ.

Two independent positive controls for actively dividing parasites (oocysts) were obtained by dissecting midguts from 20 mosquitos per genotype fed with EdU at days 13–14 after a bloodmeal, followed by fixation with 4% paraformaldehyde for 20 min at room temperature. Midguts were then washed 2× in 3% BSA for 10 min and permeabilized for 1 h in 0.5% Triton X-100 in 3% BSA and then washed twice for 10 min in 3% BSA. The Click-iT chemistry reaction mix was prepared according to the manufacturer’s instructions (Invitrogen) and added to the midguts for 30 min at room temperature. These were then washed with 3% BSA and incubated overnight at 4 °C with anti-*P. berghei* HSP70 (2E6, 1:300) followed by three washes with 3% BSA and incubation for 2 h with secondary antibodies at room temperature. The following fluorescently tagged secondary antibodies were used for detection: anti-GFP rabbit polyclonal antibody conjugated to Alexa Fluor 488 and goat anti-mouse conjugated to Alexa Fluor 647 (both 1:1,000 from Life Technologies, Invitrogen). In parallel, cell nuclei were stained with Hoechst 33342 at 1:1,000 (from Life Technologies, Invitrogen). Then midguts were washed 3× in PBS and placed a coverslip on top of the slide mounted using Fluoromount-G (SouthernBiotech). All images were acquired from 20 dissected midguts using the Zeiss confocal microscope (LSM 880) with 63× amplification and processed in ImageJ.

### Salivary-gland tissue collection for RNA sequencing

Two independent experiments were done. On day 16 post-bloodmeal, mosquitos were divided into smaller cages holding ~30–40 mosquitos. On day 19, mosquitos were either maintained in the 12 h light/dark schedule (LD) or kept in constant darkness (DD) for salivary gland collection that started on day 20 every 4 h for 2 days (experiment 1) or on day 21 for 3 days (experiment 2). When combining both experiments, for each dataset (LD or DD) the first 48 h (13 timepoints) have two independent experiments, each timepoint with a pool of salivary glands from ~20 mosquitos. The additional 24 h (6 timepoints) have one replicate of pooled salivary glands (~20 mosquitos). Each dissected mosquito showed ~30,000–50,000 sporozoites per mosquito. Mosquito euthanasia was achieved by CO_2_ to avoid a putative cold shock response of gene expression. Dissections of salivary glands were performed in PBS with RNAse inhibitor (Invitrogen) (0.1 U μl^−1^) to avoid a nutrient-rich environment that could reset the circadian rhythm of cells, and each dissected tissue was snap frozen with a minimum volume of PBS. RNA was extracted from whole salivary glands using TRIzol liquid sample according to the manufacturer’s instructions (Life Technologies). Measurements were taken from the samples discussed in this paragraph.

### Transcriptome sequencing and data analysis

Total RNA (1–2.5 μg) was enriched for mRNA using 50 μl of poly(A) beads for transcriptome sequencing according to the manufacturer’s instructions (Invitrogen). The integrity of RNA and removal of ribosomal RNAs was confirmed on a TapeStation (Agilent Technologies). Sequencing libraries were constructed using the TruSeq RNA sample preparation protocol (Illumina). Quantification of library concentration was achieved by Qubit fluorometric quantitation (Thermo Fisher Scientific), and the size of the library was calculated by TapeStation. RNA sequencing of libraries was performed on the Hiseq2000 (Illumina) with 50-bp reads for the first experiment 0–48 h LD and DD, and on the NextSeq 550 (Illumina) with 75-bp reads for the second experiment 0–72 h LD and DD, according to the manufacturer’s instructions by the UTSW McDermott Next Generation Sequencing Core. Read quality was assessed using the FASTQC quality control tool (http://www.bioinformatics.babraham.ac.uk/projects/fastqc). Reads were mapped with STAR^82^ following Cutadapt trimming^83^ to the following genomes: PlasmoDB-26_PbergheiANKA from www.PlasmoDB.org and Anopheles-stephensi-SDA-500_ AsteS1.6 from www.vectorbase.org. In the infected salivary glands, an average of 11% of the uniquely identified reads mapped to the parasite. Due to being evolutionarily distant, only 0–1% of the uniquely mapped reads mapped to both genomes. The number of reads mapping to each gene was determined and then normalized to reads per kilobase of transcript per million mapped reads (RPKM).

In this study, we generated two independent datasets for infected salivary glands in LD and DD conditions. The results presented are an average of both experiments, but analysing the individual experiments provided similar observations (Extended Data Fig. 1). To exclude that the variability between pooled samples or timepoints could influence circadian analysis owing to different numbers of sporozoite reads sequenced in each sample, that is, how infected each pool of salivary glands was, we ran the downstream analysis both with total uniquely mapped reads and with the same datasets downsampled to the lowest uniquely mapped reads. This approach allowed us to eliminate fluctuations of read number mapping to either species across timepoints. Both analyses showed the same results (Extended Data Fig. 5), further supporting that the daily rhythms observed in gene expression of both salivary glands and sporozoites were not a consequence of infection load variation in each timepoint.

For further quality control of salivary gland dissection, the obtained gene expression from these datasets (LD and DD) was compared with the defined fat body transcriptome. With www.vectorbase.org, fat body genes were considered fat body specific if genes had a fold change of 10 or greater compared with other tissues (adult female fat body sample and the reference samples were adult female Malpighian tubules, midgut and ovary)^84^. The result was 489 protein-coding genes with a fold change ≥10. Out of these 489 upregulated fat body genes, 443 genes were shown to be expressed in our dataset. To determine the relative expression of these 443 upregulated fat body genes in our dataset, the mean gene expressions of all genes across all timepoints in the LD and DD salivary gland sample (our dataset) were normalized by the maximum gene expression value of the same dataset. Similarly, all expressed genes with a transcript per million value > 1 in the VectorBase dataset were normalized to their maximum gene expression. This analysis further supported that the dataset we analysed is salivary gland specific, where the top 100 expressed genes for both datasets were plotted and compared (Extended Data Fig. 2b). Downstream bioinformatic analysis after RPKM calculations was performed in RStudio. Hierarchical clustering and heat maps were obtained for each dataset by reordering the timepoints according to gene expression using function heatmap.2 from the gplots package^85^ (Extended Data Figs. 1 and 5). Both hierarchical clustering analysis and heat maps of Spearman correlations were performed on centred log_2_-transformed RPKM values.

### Time-series analysis for circadian cycling

A gene was considered expressed if its mean expression, across all timepoints and conditions, was higher than 0.1 RPKM for the mosquito and higher than 0.5 RPKM for the parasite. With this definition, 11,383 genes, 83.6% of the mosquito genome, were identified as expressed in salivary glands; from sporozoites in salivary glands, 4,670 of the genes, 88.9% of the parasite genome, were expressed. Cycling of mRNA was assessed using four circadian statistical algorithms within the MetaCycle package^86^, which implements Lomb-scargle, JTK_CYCLE^87^, ARSER^88^ and the RAIN methods^89^. A gene was considered cycling if two of the four programs detected significant periodic expression with a threshold of *P* ≤ 0.05 and that in one of them a false discovery rate (FDR)-adjusted *P* value was ≤0.05. Phase reported by ARSER was used for further analyses. The full table of all expressed genes and their cycling analysis is reported for each species and condition in Supplementary Tables 1b–e. The circadian fold change was calculated by dividing the mean maximum expression across the 3 days of collection by the mean minimum expression across those days.

### Permutation test

To determine the number of genes that were identified as cycling by chance, we analysed 1,000 permutations of all cycling genes given by the reordering of the sampled timepoints of collection and its corresponding *Plasmodium* and *Anopheles* gene expression in LD as well as DD. Using the MetaCycle R package, each permutation was analysed for cycling genes using the JTK_CYCLE, Lomb-Scargle and ARSER algorithms. Each gene was labelled as cycling or non-cycling based on the combined *q* value of all three algorithms using Fisher’s method and defined a cycling gene with a combined *q* value of ≤0.05 for both the permutated timepoints and the real sampling order, two-tailed.

### Functional analysis

To identify the genes that cycle at a transcriptional level, gene orthologues from *Anopheles gambiae* were obtained using Biomart from www.vectorbase.org. Overall, the functions of mosquito salivary cycling genes were analysed by Kyoto Encyclopedia of Genes and Genomes (KEGG) pathway enrichment and InterPro protein domain enrichment using DAVID^90^. The top ten most significant molecular pathways sorted by fold enrichment in both environmental conditions (LD and DD) are represented (Extended Data Fig. 2c–f) and reported in detail in Supplementary Table 1g. To acquire a time-of-day resolution of functions, mosquito cycling genes were divided into 4-h-resolution phase groups and analysed for KEGG pathway and InterPro using DAVID. The most significant pathways or protein domains enriched in both environmental conditions (LD and DD) are reported for each of the 4-h intervals. No significant enrichment overlap among conditions was found for 8–12 h, 16–20 h or 20–24 h for KEGG pathways (Fig. 2). *Plasmodium berghei* functional analysis was assessed for all cycling genes owing to apparent peak expression in the first 4 h of the day, in both conditions (LD and DD). The GO term for biological process and KEGG pathway analysis were performed in www.PlasmoDB.org (Fig. 4 and Extended Data Fig. 7) and are reported in detail in Supplementary Table 1g. By overlapping our dataset of cycling mRNAs with the Lindner 2013 dataset of sporozoite proteins expressed in the salivary gland^77^, we identified 238 genes that cycle at the mRNA level and whose proteins are expressed during the sporozoite stage in the salivary glands (Extended Data Figs. 6b and 8).

### Proteomics analysis

#### Protein extraction

Infected salivary glands were collected from female *A. stephensi* mosquitos and snap frozen. For each timepoint (ZT0, 6, 12 and 18), three replicates were collected where each replicate had five pairs of infected salivary glands. Fifty microlitres of fresh RIPA protease inhibitor cocktail was added to each sample pellet, Piece RIPA Buffer (Thermo Fisher Scientific), 1× Halt Protease & Phosphatase Inhibitor Cocktail (100×) (Thermo Fisher Scientific) and 1× 0.5 M EDTA solution (100×) (Thermo Fisher Scientific), and samples were homogenized with micropestles. Samples were then vortexed and incubated on ice for 10 min to facilitate lysis. The salivary gland homogenate suspension sample was spun in a microcentrifuge for 20 min for 13,800*g* at 4 °C. The protein supernatant was transferred, and protein was quantified using the Pierce BCA Protein Assay Kit (Thermo Fisher Scientific). Preparation of diluted standards and sample preparations were done according to the manufacturer’s instructions. In brief, each protein lysate sample was diluted at 1:2 in the RIPA protease inhibitor cocktail. Then, 200 μl working reagent was added to each sample well and shaken for 30 min with a plate shaker. The 96-well plate was then incubated for 30 min at room temperature. The absorbance was measured at 562 nm using the CLARIOstar plate reader. A linear regression equation and standard curve were calculated from the absorbance values of these diluted standard duplicates using Prism.

#### Peptide preparation with S-Trap microcolumns

A solution of Tris (pH 8) and sodium dodecyl sulfate (SDS) in water was added to sample lysates to achieve a final concentration of 5% SDS, 50 mM Tris in 57.5 μl total sample volume. For the reduction and alkylation of cysteines, the samples were incubated at 37 °C, 1,200 rpm with 5 mM dithiothreitol (Pierce) for 1 h, then 14 mM iodoacetamide (Pierce) for 45 min. Subsequent binding and washing of the samples on S-Trap micro columns (Protifi) were performed following the manufacturer’s protocol. For protein digestion, 50 mM tetraethylammonium bromide containing trypsin (Pierce) and Lys-C (FUJIFILM Wako Pure Chemical Corporation) was added to the S-Trap column and incubated at 47 °C for 1.5 h. Each protease was used at a protease to protein ratio of 1:20 (w/w) or minimum of 0.5 μg. Digested peptides were sequentially eluted with 50 mM tetraethylammonium bromide, 0.2% formic acid (FA) and 50% acetonitrile. The resulting eluates were dried in a SpeedVac and resuspended in 0.1% FA, 0.015% *n*-dodecyl-β-d-maltoside. Peptide concentrations were measured by NanoDrop, and the samples were further diluted in 0.1% FA, 0.015% *n*-dodecyl-β-d-maltoside to a final concentration of 10 ng μl^−1^.

#### LC–MS/MS data acquisition

Samples were analysed on a trapped ion mobility spectrometry (tims), detected in quadropole time of flight (TOF), single-cell proteomics (SCP) instrument (timsTOF SCP) (Bruker Daltonics) coupled to an Evosep One liquid chromatography (LC) system (Evosep Biosystems). Samples were prepared for injection by loading 20 μg of peptides each onto Evotip Pures (Evosep Biosystems). Peptides were eluted online from the Evotips and separated by reverse-flow chromatography on a 15-cm PepSep column (75-μm internal diameter, 1.9-μm C18 beads; Bruker Daltonics) with a 10-μm zero-dead volume sprayer (Bruker Daltonics), using the Whisper 20 method (60-min gradient length, 100 nl min^−1^ flow rate) from Evosep. The column was maintained at 50 °C in a Bruker column toaster.

Data were acquired in data-dependent acquisition with parallel accumulation serial fragmentation (dda-PASEF) mode with high sensitivity detection enabled. Ions were delivered to the timsTOF SCP through CaptiveSpray ionization and analysed across a mass range of 100–1,700 *m*/*z* and mobility range of 0.7–1.3 1/*k*_0_ (*k*_0_ is reduced ion mobility, which is a standard measure of how ions move through a gas buffer, adjusted for temperature and pressure). Accumulation and ramp time were both set to 166 ms, with a 100% duty cycle. For each mass spectrometry (MS)1 scan, five MS2 PASEF ramps were performed for a total cycle time of 1.03 ms. A polygon filter was used to exclude singly charged precursor species. Quadrupole isolation of precursors for MS2 fragmentation was set to allow a 2 *m*/*z* window for precursors under 700 *m*/*z* and a 3 *m*/*z* window for precursors above 800 *m*/*z*. Collision energy linearly increased with ion mobility, from 20 eV at 0.6 1/*k*_0_ to 65 eV at 1.6 1/*k*_0_. The intensity threshold for precursor repetitions was set to 500, with a target intensity of 20,000. Active exclusion of precursors was released after 0.2 min.

#### Raw data searching and data analysis

The raw LC–MS/MS data were searched with Fragpipe 20.0 against a combined *A. stephensi* SDA-500 (www.vectorbase.org) and *P. berghei* ANKA database (www.PlasmoDB.org), with contaminant and reverse sequences added by Fragpipe. Redundant protein sequences were consolidated into single FASTA entries. Entries were reformatted to allow Fragpipe to parse out relevant information, including protein ID and description, organism and gene name. Default parameters for closed, tryptic search were used, with the inclusion of deamidation (NQ) and phosphorylation (STY) as additional variable modifications. MS1 quantification was also performed, without match-between-runs and normalization across runs enabled. The MSstats.csv output generated by Fragpipe was used for downstream analyses. Features mapping to contaminant proteins were removed before data processing with the MSstats R package. The dataProcess function was run with default parameters, which performs log_2_ intensity transformation, normalization by equalizing medians, run-level protein intensity summarization and imputation using an accelerated failure time model. Significance testing and fold-change calculation between conditions was performed using the MSstats function groupComparison (Supplementary Table 1f).

#### Saliva proteomics

Infected female *A. stephensi* mosquitoes were starved for 10–14 h before a forced salivation assay performed at ZT4 and at ZT16. Mosquitoes were anaesthetized on ice, mosquitos’ legs and wings were removed and their bodies were superglued onto a slide on its right side. Mosquitoes were oriented in a circle formation, with proboscises in a droplet of 10 μl of 1× PBS pH 7.2. Salivation occurred at 21 °C at 80% humidity for 10–15 min, in groups of three to six mosquitoes per glass slide. A total of 18–47 mosquitoes were utilized for each independent experiment, with 3 independent experiments (replicates) per timepoint. For the ZT16 timepoint, experiments were done in the dark and using a red lamp as a light source. After collection, saliva samples were placed into a −80 °C freezer until protein extraction. Protein extraction and MS were as described above.

### Quantification of mosquitos’ bloodmeal

#### Mosquito biting

Mosquitos were reared in two incubators kept in opposite light/dark cycles. Mosquitos were transferred to small cups containing between 5 and 20 mosquitos each and fasted for 12 h before the biting experiment. Mosquitos in each cup were allowed to feed either on one anaesthetized mouse or through membrane feeding on human blood for these experiments. When allowed to bite on anaesthetized mice at two times of day—at ZT4 (4 h after lights on) and at Z16 (4 h after lights off)—mosquitos had access to the mouse for 30 min in their respective light settings. For membrane feeding, the glytube protocol was adapted^91^. For assembling the heating element of the glytube, heat-resistance plastic sealing film was soldered onto the 50-ml conical tube to create a seal and prevent glycerol leakage. Two layers of stretch parafilm 5 cm × 5 cm were stretched over the heat-resistance plastic sealing film. Glytubes were prewarmed overnight or for a minimum of 30 min before the feeding experiment at 50 °C. One millilitre of washed human blood (red blood cells, BioIVT) was prewarmed for 15–30 min at 37 °C and placed onto the cap reservoir with stretched parafilm for mosquito feeding. The inverted glytubes were held in place on top of the cage mesh by a metal clamp stand for mosquitos to feed for 30 min in their respective light settings.

Mosquitos were considered successful in achieving a bloodmeal by observing the mosquito abdomen for enlargement and presence of blood during midgut dissection using a light microscope. To obtain the percentage of mosquito biting, the total number of mosquitos with a bloodmeal was divided by the total number of mosquitos in the cup (an independent experiment). The behavioural assay was performed six independent times for both daytime and nighttime timepoints.

#### Mosquito blood ingestion

Midguts of each mosquito were dissected into 100 μl HBSS (Gibco) and immediately frozen. On analysis day, samples were thawed and homogenized with a pestle. Samples were spun at 2,800*g* for 8 min, the supernatant was collected in a 96-well plate and haemoglobin colorimetry assessment was performed as recommended by the manufacturer (Invitrogen). In brief, diluted high-sensitivity standards were prepared and the samples were incubated with the diluent for 30 min at room temperature. Haemoglobin colorimetry was measured using plate reader absorbance at 562 nm at room temperature. A standard curve using two replicates of raw diluted standard absorbance values was calculated using a linear regression equation generated from Prism and/or by the MARS software from the ClarioStar plate reader. The linear regression equation was used to calculate the haemoglobin concentration (mg ml^−1^) from raw sample absorbance values. ZT4 and ZT16 feeding experiments done on the same day were analysed together in the haemoglobin colorimetry assay, where each time a new standard curve was generated from diluted standards via linear regression analysis. Sample haemoglobin concentrations were calculated from a linear regression equation generated from each standard curve.

### Parasite load in mouse liver

*Anopheles stephensi* mosquitos infected with *P. berghei* ANKA-GFP (259cl2) were maintained in 12 h light/12 h dark cyclic conditions.

From mosquito bite, 24 mice (C57BL/6, male, 9–12 weeks of age) were anaesthetized with 90 mg kg^−1^ of ketamine and 5 mg kg^−1^ of xylazine. Each mouse was placed ventral side down on a mesh-covered enclosure containing 15–20 female *A. stephensi* mosquitos. At ZT4 (4 h after lights on, when mosquitos do not typically bite) and at Z16 (4 h after lights off, close to natural biting time of *A. stephensi*) 21 days after mosquito’s bloodmeal, mosquitos were allowed to feed freely for 25 min at the appropriate light, temperature and humidity to the mosquito time of day. Livers were collected 46 h after the midpoint of infection and snap frozen. The whole tissue was homogenized in 4 ml of TRIzol (Thermo Fisher Scientific), one-fourth processed according to the manufacturer’s instructions. cDNA synthesis was performed with 500 ng of purified RNA using the UltraScript cDNA Synthesis Kit (Genessee ScientificNZYTech), as per the manufacturer’s instructions. cDNA was then used for quantitative polymerase chain reaction (qPCR) using PowerTrack SYBR Green (ThermoFisher) with the pair of primers for mouse glyceraldehyde-3-phosphate dehydrogenase (*mGapdh*: CAA GGA GTA AGA AAC CCT GGA CC; CGA GTT GGG ATA GGG CCT CT) and parasite 18S ribosomal RNA (*Pb18S rRNA*: AAG CAT TAA ATA AAG CGA ATA CAT CCT TAC; GGA GAT TGG TTT TGA CGT TTA TGT G). Measurements of SYBR fluorescence were performed on Bio-Rad CFX96 C1000 Real-Time PCR Systems (Bio-Rad Laboratories) and analysed using the 2^−ΔΔCt^ method.

From sporozoite injection, three independent experiments were performed. Twenty-one days after the mosquitos’ bloodmeal, to ensure the sporozoites were fully mature, salivary glands from ~50 infected mosquitos at ZT4 (4 h after lights on, when mosquitos do not typically bite and close to the peak of sporozoite gene expression) and at Z16 (4 h after lights off, close to natural biting time of *A. stephensi*) were collected into basal DMEM (Gibco) medium. Salivary glands were ground with a plastic pestle and filtered through a 40-μm Falcon cell strainer (Thermo Fisher Scientific) to release sporozoites. Sporozoites were then counted using a haemocytometer (Marienfeld Superior) followed by intradermal injection in the left hind leg of each mouse with 30,000 sporozoites in 50 µl of DMEM. Four experimental groups were designed, with 10–23 mice (C57BL/6, male, 6 weeks of age) per group: ZT4-matched sporozoites and mice, ZT16-matched sporozoites and mice, and two mismatched groups, meaning that sporozoites collected from ZT4 mosquitos were injected in mice at ZT16, and the complementary group. Thirty-two hours after injection, livers were collected and homogenized in 3 ml of TRIzol (Thermo Fisher Scientific) containing 0.1 mm zirconia/silica beads (BiospecTM Products). Liver homogenization was performed through mechanical disruption in a Mini-BeadBeater (BioSpec Products) for 1.5 min. The 3 ml of homogenized tissue were divided into three clean 1.5-ml microcentrifuge tubes (Eppendorf) each with 1 ml of tissue lysate. One aliquot of 1 ml was used for extraction of total RNA by addition of 200 µl of chloroform, followed by a 4 °C centrifugation at 12,000*g* for 15 min. The upper aqueous phase was then transferred to a new microcentrifuge tube, and RNA extraction proceeded according to the manufacturer’s instructions of the NZY Total RNA Isolation Kit (NZYTech). Quantification of total RNA concentration was performed on NanoDrop 2000 spectrophotometer (Thermo Fisher Scientific) according to the manufacturer’s guidelines. cDNA synthesis was performed with 1 µg of purified RNA using the NZY First-Strand cDNA Synthesis Kit (NZYTech), as per the manufacturer’s instructions. cDNA was then used for qPCR using iTaq Universal SYBR Green Supermix (Bio-Rad Laboratories) with the pair of primers for mouse hypoxanthine-guanine phosphoribosyltransferase (*mHprt*: CAT TAT GCC GAG GAT TTG GA; AAT CCA GCA GGT CAG CAA AG) and parasite 18S ribosomal RNA (*Pb18S rRNA*: AAG CAT TAA ATA AAG CGA ATA CAT CCT TAC; GGA GAT TGG TTT TGA CGT TTA TGT G). Measurements of SYBR fluorescence were performed on ViiA 7 (384-well plates) Real-Time PCR Systems (Thermo Fisher Scientific).

### Parasite motility

For gliding assays, 50,000 sporozoites from salivary glands of mosquitos were dissected in the morning (ZT4) or the evening (ZT16). Sporozoites were incubated in complete DMEM medium at 37 °C on glass coverslips. After 30 min of incubation, sporozoites were fixed and stained with αCSP (kindly provided by Miguel Prudêncio), αTRAP antibodies (kindly provided by Joana Tavares) and Hoechst (DNA dye). Coverslips were imaged using a Zeiss 880 confocal microscope, and sporozoite motility was quantified by counting the number of circles of CSP and TRAP formed by each individual sporozoite. An average of 40 sporozoites were imaged per replicate per time of the day. The number of sporozoite trails was quantified by three independent experimenters from images of fixed and stained sporozoites^92^.

### Hepatocyte reporter line and infections

Hepa 1-6 cells were kindly provided by the Arruda laboratory (University of California, Berkeley) and cultured in complete DMEM high glucose, GlutaMAX (Fisher Scientific) supplemented with 10% foetal bovine serum (Fisher Scientific) and 1% penicillin–streptomycin (Fisher Scientific). Cells were maintained at 37 °C, 5% CO_2_ in a standard cell culture incubator. Hepa 1-6 cells expressing *Bmal1* promoter luciferase were generated via lentiviral transduction as previously described^93^. For experiments, cells were seeded in a 96-well plate (25,000 cells per well) and let attach at 37 °C overnight. For Hepa 1-6 synchronization, plates were then incubated at alternating 37 °C/35 °C for 2 days in the DigiTherm CO_2_ Heating/Cooling Incubator (Tritech Research), while non-synchronized cells were maintained at constant 37 °C. To record *Bmal1* promoter luciferase, medium was supplemented with 100 μM luciferin (VivoGlo, Promega) and luciferase activity was recorded at 30-min intervals using a CLARIOstar luminometer (BMG Labtech), where cells were kept at alternating 37 °C/35 °C and 5% CO_2_.

Salivary glands from infected mosquitos (days 21–24 post-bloodmeal) were dissected into HBSS (Fisher Scientific) during the mosquitos’ day (light on, ZT0–4) or night (light off, ZT12–16). Sporozoites were released and counted with a haemocytometer, 12,500 sporozoites were added per well and plates were spun down for 5 min (ref. ^93^). Two hours later, the medium was changed to complete DMEM supplemented with 50 μg ml^−1^ gentamicin (Gibco) and 1 μg ml^−1^ fungizone (Sigma Aldrich). Cells were lysed 48 h after infection, RNA was extracted using the RNeasy kit (QIAGEN) following the manufacturer’s instructions and reverse transcription was performed using a cDNA synthesis kit (Genesee Scientific). qPCR was performed using PowerTrack SYBR Green Master Mix (Fisher Scientific) in Bio-Rad CFX96 C1000 Real-Time PCR Systems (Bio-Rad Laboratories) with the following primers: mouse glyceraldehyde-3-phosphate dehydrogenase and parasite 18S ribosomal RNA. mRNA expression was calculated relative to *Gapdh* expression using the 2^−ΔΔ^Ct method. Statistical analysis was performed using Prism 10.

### Reporting summary

Further information on research design is available in the Nature Portfolio Reporting Summary linked to this article.

## Supplementary information

Reporting Summary

Supplementary Data

## Source data

Source Data Fig. 1 Statistical source data.

Source Data Fig. 2 Statistical source data.

Source Data Fig. 3 Statistical source data.

Source Data Fig. 4 Statistical source data.

Source Data Extended Data Fig. 1 Statistical source data.

Source Data Extended Data Fig. 2 Statistical source data.

Source Data Extended Data Fig. 3 Statistical source data.

**Extended Data Fig. 4:**
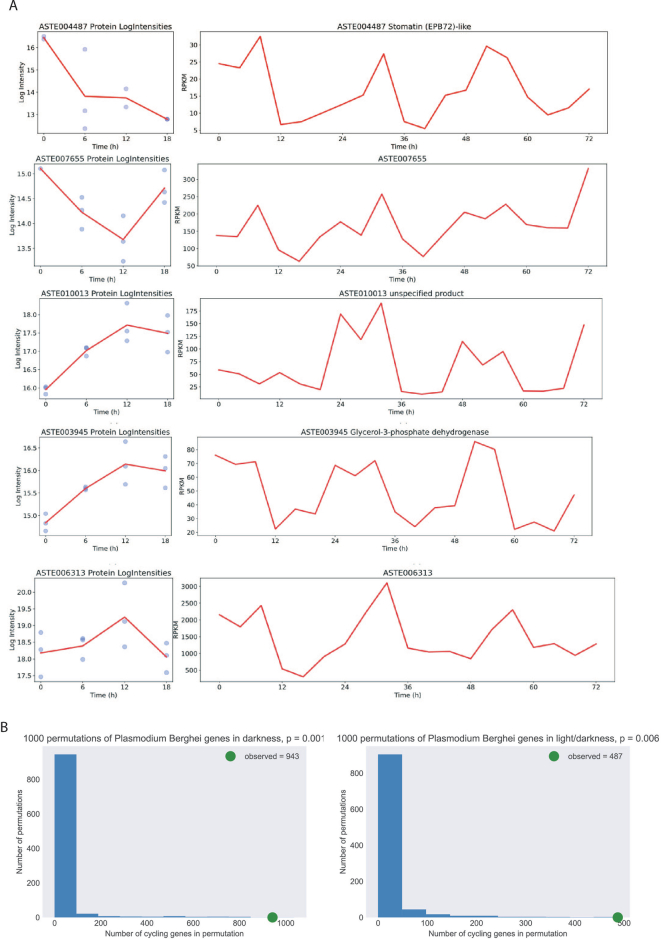
Cycling genes and proteins in mosquito salivary glands and Plasmodium sporozoites. **A**. Salivary gland protein expression and transcript expression profiles across time for some DE proteins. Left plots: Mean log2 Intensities (red line) of proteins DE across time. Each blue point represents log2 intensity value for one of three replicates. Right plots: For all proteins plotted, their transcripts cycle throughout the day. Bottom-most protein and transcript is a blood-meal related gene. **B**. Histograms showing that across 1,000 permutations of Plasmodium genes in both light/darkness and complete darkness over 72 h, no permutations had more cycling genes than the observed sample (green dot), as determined by a combination of cycle detection algorithms. This analysis showed that on average, only 22 genes would be recognized as cycling by chance, whereas the real time point order identified between 500 to 950 cycling genes. Source data

Source Data Extended Data Fig. 4 Statistical source data.

**Extended Data Fig. 5:**
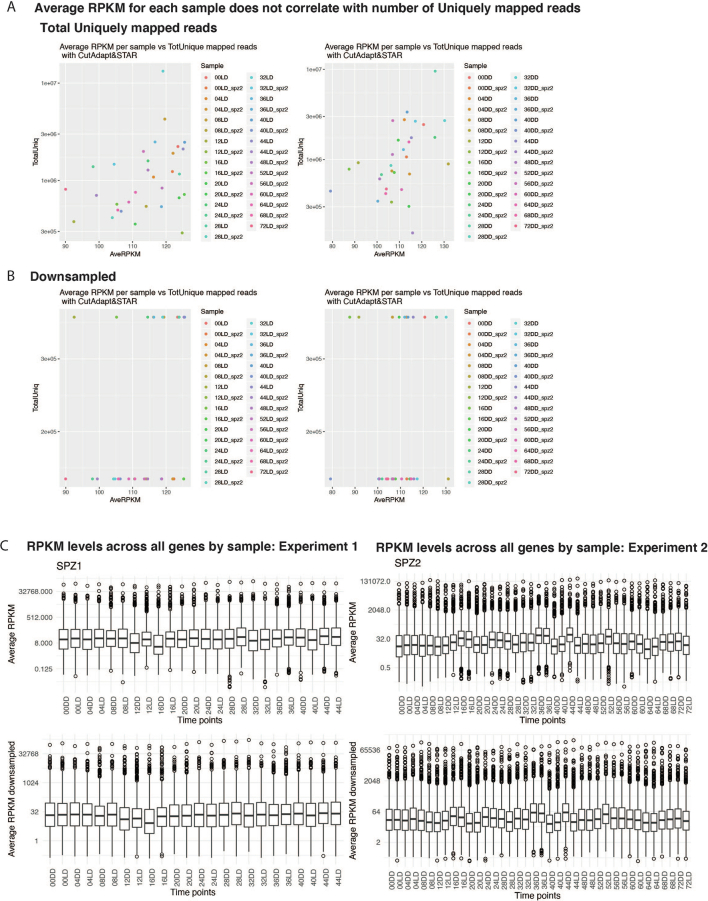
Quality analysis of unique mapped genes. **A**. Quality control of reads per kilobase per million (RPKM) versus number of uniquely mapped reads obtained across time points. **B**-**C**. Downsampling of uniquely mapped reads to normalize for potential fluctuation in parasite numbers across time points, show similar findings. Represented results are from two independent experiments (left: experiment 1; right: experiment 2). Source data

Source Data Extended Data Fig. 5 Statistical source data.

**Extended Data Fig. 6:**
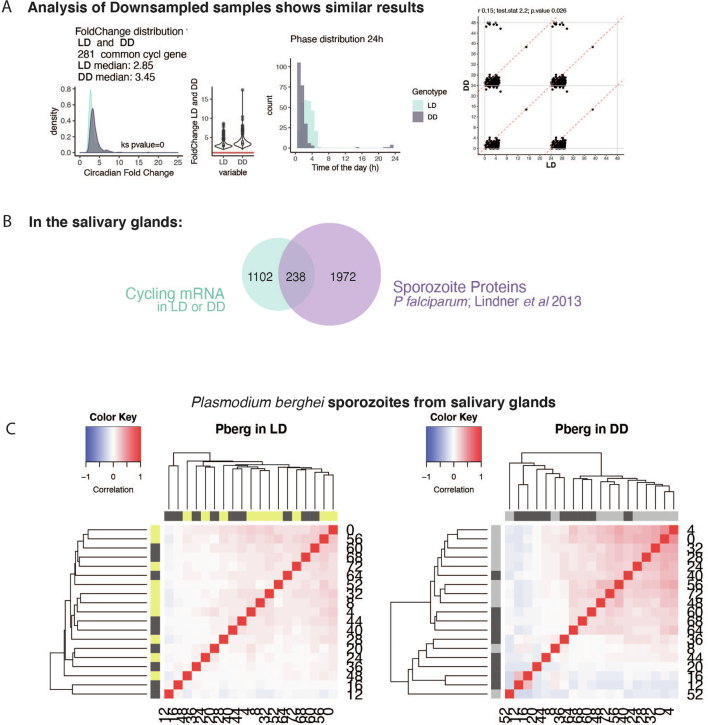
*Plasmodium berghei* sporozoite stage shows transcriptional rhythms. **A**. Re-analysis of both circadian cycling genes and GO-term enrichment using the downsampled datasets brings the reads of each time point to the same number, removing the potential confounder of variable number of parasites in the salivary glands, and shows the same results as the actual sequenced reads. **B**. Venn diagram representing the common cycling mRNAs in LD or DD identified from our dataset that were also shown to be previously identified sporozoite proteins from Plasmodium falciparum in Linder et al., 2013. **C**. Hierarchical clustering of samples in LD and DD. Source data

Source Data Extended Data Fig. 6 Statistical source data.

**Extended Data Fig. 7:**
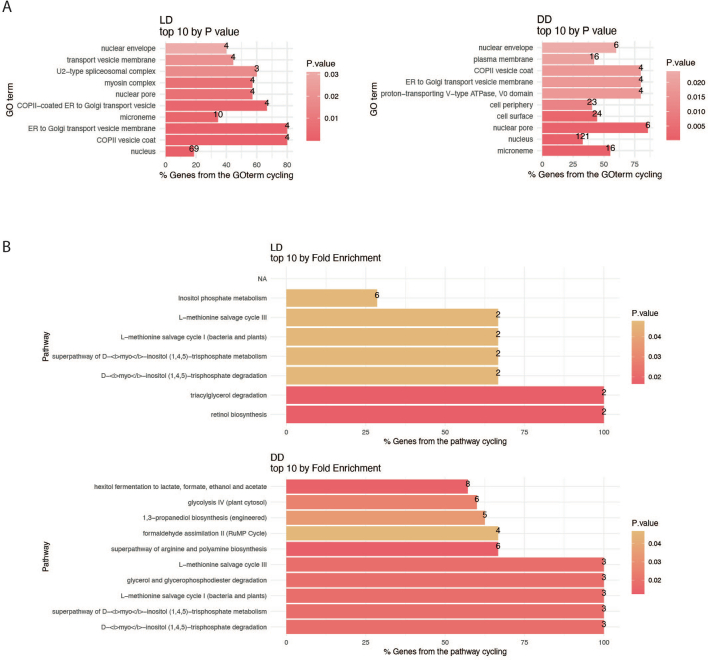
The most significant biological functions of cycling sporozoite genes in DD and LD. **A**. Cycling genes’ top 10 enriched GO terms in LD and DD conditions. **B**. Cycling genes’ top 10 enriched KEGG pathways sorted by fold enrichment in LD and DD conditions. Source data

Source Data Extended Data Fig. 7 Statistical source data.

**Extended Data Fig. 8:**
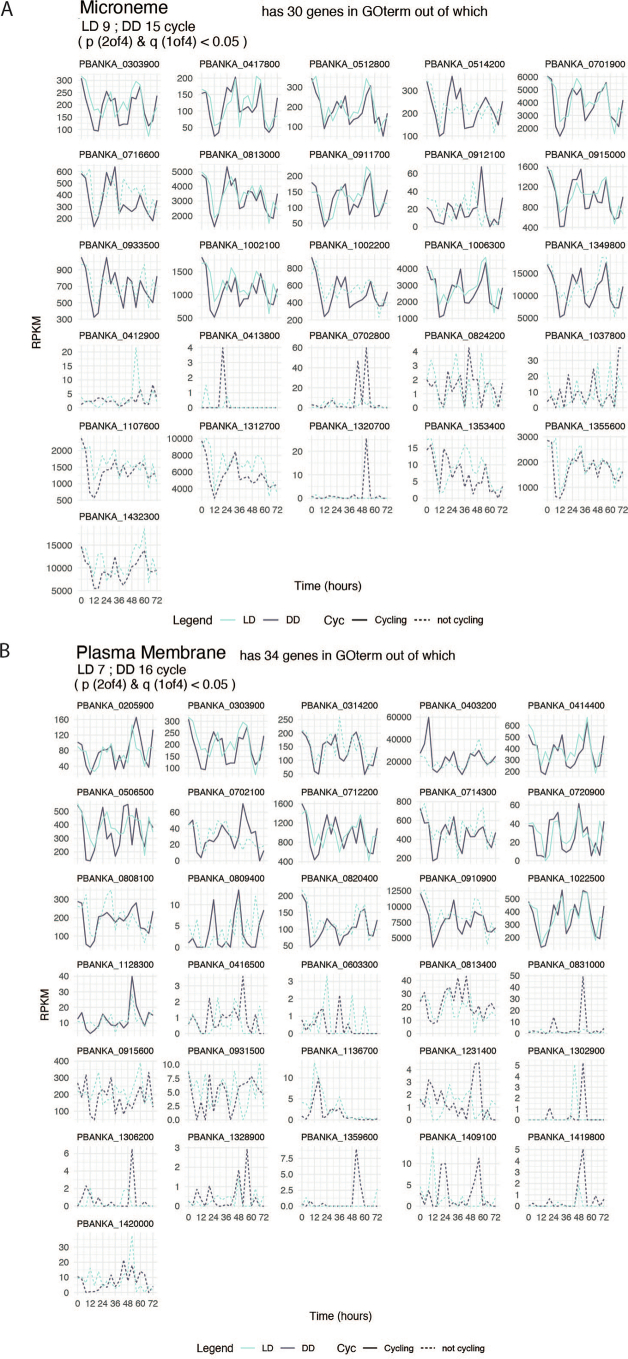
Profile of expression of genes within the enriched GO term. **A**. Representation of microneme associated genes. **B**. Representation of plasma membrane associated genes. Source data

Source Data Extended Data Fig. 8 Statistical source data.

**Extended Data Fig. 9:**
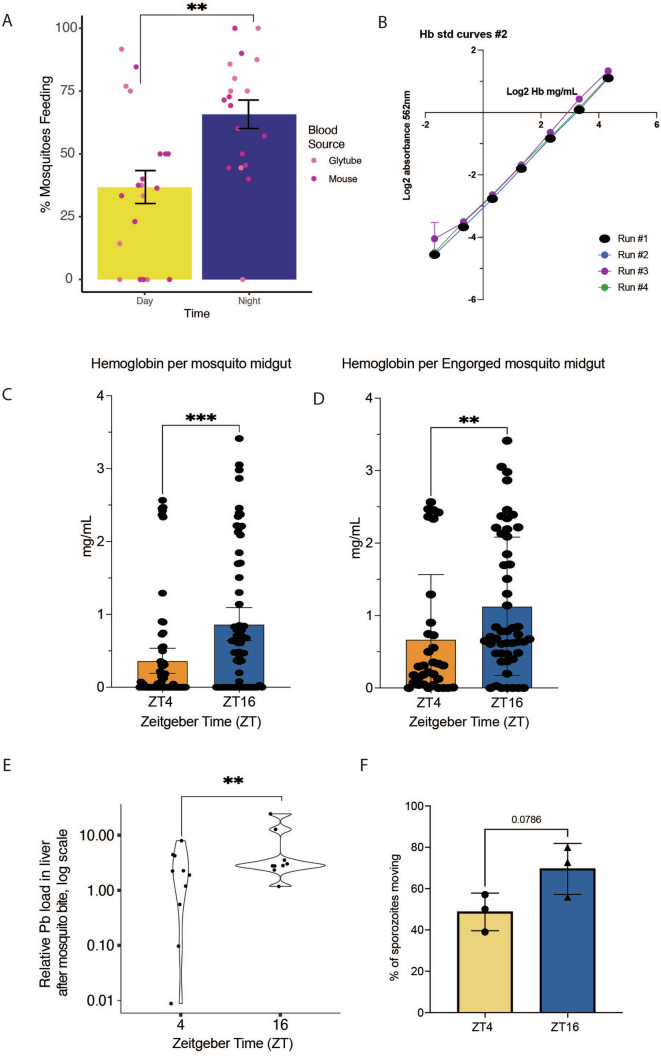
Night period dictates mosquitos efficient feeding behaviour and sporozoite increased motility. **A**. Percent of mosquito biting across two times of the day (ZT4 and ZT16) on anesthetized mice or warm human blood was significantly higher at night (ZT16) compared to daytime (ZT4). Each point represents one replicate from 8 independent experiments, where 5-20 co-housed mosquitoes had access to a bloodmeal from one of two sources mentioned above. Percent values were derived from dividing the total number of fed mosquitoes that were co-housed by the total number of mosquitoes that were co-housed. Two-way ANOVA, adjusted for multiple comparisons ** p = 0.00257. There was no difference in percent of mosquito biting on different blood sources during the same time of day (Two-way ANOVA, p = 0.4622). Bars represent mean and error bars represent +/- SEM. **B**. Four independent normalized hemoglobin standard curves plotted from corresponding linear regression equation calculated from the absorbance (562 nm) values of diluted hemoglobin standards from four independent runs of the hemoglobin colorimetric assay. Individual points represent mean log2 absorbance of two technical replicates per independent run. Data are presented as mean values +/- SD. **C**. Quantification of hemoglobin in each mosquito midgut, as a proxy of blood ingested, after mosquito access to a bloodmeal for 30 min (n = 67 mosquito midguts for daytime group and n = 68 mosquito midguts for nighttime group; 4 independent experiments with 2 replicates per timepoint of 5-20 mosquitoes feeding on blood source; Mann Whitney test, *** p = 0.001, two-tailed). Hemoglobin concentrations (mg/mL) of all mosquitos from glytube feeding experiments, regardless of blood-fed status. Hemoglobin levels were quantified from linear regression equation generated from hemoglobin standard curves. Data are presented as mean values with 95% confidence intervals. **D**. Quantification of hemoglobin in each engorged mosquito midgut, as a proxy of blood ingested, after mosquito access to a bloodmeal for 30 min (n = 33 engorged midguts for daytime group; n = 50 engorged midguts for nighttime group; 4 independent experiments). Hemoglobin concentrations (mg/mL) of only blood-fed mosquitos from glytube feeding experiments. Quantified from linear regression equation generated from hemoglobin standard curves. Data are presented as mean values +/- SEM. **E**. Parasite load in livers of mice after bite of infected mosquitos at ZT4 or ZT16. Quantification of parasite load by qPCR analysis of Plasmodium berghei 18S expression in the livers of mice at 46 h post infection by bite of infected mosquitos for 25 min (n = 24 mice from 4 independent experiments; t test, ** p = 0.007). **F**. The percentage of sporozoites moving were quantified at each time of the day (ZT4 and ZT16) by taking the sum of sporozoites that moved in 1-5 trails, 6-10 trails, or > 10 trails category and dividing it by the total number of sporozoites imaged for each timepoint in 3 replicates (total 3 replicates) per timepoint. For ZT4, n = 143 sporozoites across all replicates. For ZT16, n = 128 sporozoites across all replicates. An average of 40 sporozoites were imaged per replicate per time of day. Statistical analyses were performed in prism using the student’s t-test. Data are presented as mean values +/- SD. Source data

Source Data Extended Data Fig. 9 Statistical source data.

## Data Availability

All data are available in the extended data and in the source data for each figure. All sequencing data, raw and processed, are deposited in Gene Expression Omnibus datasets (www.ncbi.nlm.nih.gov/geo/query/acc.cgi?acc=GSE284425). Source data are provided with this paper.

## Code availability

The code used is available from the packages mentioned in Methods.

## References

[1] *World Malaria Report 2022* (WHO, 2022).

[2] Rund, S. S., O’Donnell, A. J., Gentile, J. E. & Reece, S. E. Daily rhythms in mosquitoes and their consequences for malaria transmission. *Insects* **7**, 14 (2016). 27089370 10.3390/insects7020014 PMC4931426

[3] Jones, M. D., Ford, M. G. & Gillett, J. D. Light-on and light-off effects on the circadian flight activity in the mosquito *Anopheles gambiae*. *Nature* **211**, 871–872 (1966). 5968773 10.1038/211871b0

[4] Sheppard, A. D. et al. Light manipulation of mosquito behaviour: acute and sustained photic suppression of biting activity in the *Anopheles gambiae* malaria mosquito. *Parasit. Vectors* **10**, 255 (2017). 28619089 10.1186/s13071-017-2196-3 PMC5472875

[5] Das, S. & Dimopoulos, G. Molecular analysis of photic inhibition of blood-feeding in *Anopheles gambiae*. *BMC Physiol.* **8**, 23 (2008). 19087335 10.1186/1472-6793-8-23 PMC2646746

[6] Maxwell, C. A., Wakibara, J., Tho, S. & Curtis, C. F. Malaria-infective biting at different hours of the night. *Med. Vet. Entomol.* **12**, 325–327 (1998). 9737608 10.1046/j.1365-2915.1998.00108.x

[7] Korgaonkar, N. S., Kumar, A., Yadav, R. S., Kabadi, D. & Dash, A. P. Mosquito biting activity on humans & detection of *Plasmodium falciparum* infection in *Anopheles stephensi* in Goa, India. *Indian J. Med. Res.* **135**, 120–126 (2012). 22382193 10.4103/0971-5916.93434 PMC3307172

[8] Braack, L. E. et al. Biting pattern and host-seeking behavior of *Anopheles arabiensis* (Diptera: Culicidae) in northeastern South Africa. *J. Med. Entomol.* **31**, 333–339 (1994). 8057306 10.1093/jmedent/31.3.333

[9] Pimenta, P. F., Touray, M. & Miller, L. The journey of malaria sporozoites in the mosquito salivary gland. *J. Eukaryot. Microbiol.* **41**, 608–624 (1994). 7866385 10.1111/j.1550-7408.1994.tb01523.x

[10] Frischknecht, F. & Matuschewski, K. Plasmodium Sporozoite Biology. *Cold Spring Harb. Perspect. Med.*10.1101/cshperspect.a025478 (2017). 10.1101/cshperspect.a025478 PMC5411682 28108531

[11] Ribeiro, J. M. & Francischetti, I. M. Role of arthropod saliva in blood feeding: sialome and post-sialome perspectives. *Annu. Rev. Entomol.* **48**, 73–88 (2003). 12194906 10.1146/annurev.ento.48.060402.102812

[12] Kalume, D. E. et al. A proteomic analysis of salivary glands of female *Anopheles gambiae* mosquito. *Proteomics* **5**, 3765–3777 (2005). 16127729 10.1002/pmic.200401210

[13] Arora, G. et al. Immunomodulation by mosquito salivary protein AgSAP contributes to early host infection by *Plasmodium*. *mBio* **12**, e0309121 (2021). 34903042 10.1128/mBio.03091-21 PMC8669493

[14] Arca, B. et al. An updated catalogue of salivary gland transcripts in the adult female mosquito, *Anopheles gambiae*. *J. Exp. Biol.* **208**, 3971–3986 (2005). 16215223 10.1242/jeb.01849

[15] Pala, Z. R. et al. Mosquito salivary apyrase regulates blood meal hemostasis and facilitates malaria parasite transmission. *Nat. Commun.* **15**, 8194 (2024). 39294191 10.1038/s41467-024-52502-3 PMC11410810

[16] Yoshida, S. et al. Inhibition of collagen-induced platelet aggregation by anopheline antiplatelet protein, a saliva protein from a malaria vector mosquito. *Blood* **111**, 2007–2014 (2008). 18056842 10.1182/blood-2007-06-097824

[17] Islam, A. et al. Anopheline antiplatelet protein from mosquito saliva regulates blood feeding behavior. *Sci. Rep.* **9**, 3129 (2019). 30816309 10.1038/s41598-019-39960-2 PMC6395645

[18] Chuang, Y. M. et al. *Anopheles gambiae* lacking AgTRIO inefficiently transmits *Plasmodium berghei* to mice. *Infect. Immun.*10.1128/IAI.00326-19 (2019). 10.1128/IAI.00326-19 PMC6704594 31285253

[19] Schleicher, T. R. et al. A mosquito salivary gland protein partially inhibits *Plasmodium* sporozoite cell traversal and transmission. *Nat. Commun.* **9**, 2908 (2018). 30046053 10.1038/s41467-018-05374-3 PMC6060088

[20] Klug, D., Gautier, A., Calvo, E., Marois, E. & Blandin, S. A. The salivary protein Saglin facilitates efficient midgut colonization of *Anopheles* mosquitoes by malaria parasites. *PLoS Pathog.* **19**, e1010538 (2023). 36862755 10.1371/journal.ppat.1010538 PMC10013899

[21] Dragovic, S. M. et al. Immunization with AgTRIO, a protein in *Anopheles* saliva, contributes to protection against *Plasmodium* infection in mice. *Cell Host Microbe* **23**, 523–535 (2018). 29649443 10.1016/j.chom.2018.03.008 PMC5998332

[22] Chuang, Y. M. et al. The effects of a mosquito salivary protein on sporozoite traversal of host cells. *J. Infect. Dis.* **224**, 544–553 (2021). 33306099 10.1093/infdis/jiaa759 PMC8328219

[23] Yamazaki, S. et al. Resetting central and peripheral circadian oscillators in transgenic rats. *Science* **288**, 682–685 (2000). 10784453 10.1126/science.288.5466.682

[24] Yoo, S. H. et al. PERIOD2::LUCIFERASE real-time reporting of circadian dynamics reveals persistent circadian oscillations in mouse peripheral tissues. *Proc. Natl Acad. Sci. USA* **101**, 5339–5346 (2004). 14963227 10.1073/pnas.0308709101 PMC397382

[25] Zhang, R., Lahens, N. F., Ballance, H. I., Hughes, M. E. & Hogenesch, J. B. A circadian gene expression atlas in mammals: implications for biology and medicine. *Proc. Natl Acad. Sci. USA* **111**, 16219–16224 (2014). 25349387 10.1073/pnas.1408886111 PMC4234565

[26] Borrmann, H. R.-F. F. Crosstalk between circadian clocks and pathogen niche. *PLoS Pathog.*10.1371/journal.ppat.1012157 (2024). 10.1371/journal.ppat.1012157 PMC11081299 38723104

[27] Rund, S. S. et al. Daily rhythms in antennal protein and olfactory sensitivity in the malaria mosquito *Anopheles gambiae*. *Sci. Rep.* **3**, 2494 (2013). 23986098 10.1038/srep02494 PMC3756343

[28] Rund, S. S., Hou, T. Y., Ward, S. M., Collins, F. H. & Duffield, G. E. Genome-wide profiling of diel and circadian gene expression in the malaria vector *Anopheles gambiae*. *Proc. Natl Acad. Sci. USA* **108**, E421–E430 (2011). 21715657 10.1073/pnas.1100584108 PMC3156198

[29] Wang, G. et al. Clock genes and environmental cues coordinate *Anopheles* pheromone synthesis, swarming, and mating. *Science* **371**, 411–415 (2021). 33479155 10.1126/science.abd4359 PMC9854397

[30] Spence, P. J. et al. Vector transmission regulates immune control of *Plasmodium* virulence. *Nature* **498**, 228–231 (2013). 23719378 10.1038/nature12231 PMC3784817

[31] Jones, M. D., Hill, M. & Hope, A. M. The circadian flight activity of the mosquito *Anopheles gambiae*: phase setting by the light regime. *J. Exp. Biol.* **47**, 503–511 (1967). 5592417 10.1242/jeb.47.3.503

[32] Taylor, B. & Jones, M. D. The circadian rhythm of flight activity in the mosquito *Aedes aegypti* (L.). The phase-setting effects of light-on and light-off. *J. Exp. Biol.* **51**, 59–70 (1969). 5387705 10.1242/jeb.51.1.59

[33] Rijo-Ferreira, F. & Takahashi, J. S. Genomics of circadian rhythms in health and disease. *Genome Med.* **11**, 82 (2019). 31847894 10.1186/s13073-019-0704-0 PMC6916512

[34] Rijo-Ferreira, F., Takahashi, J. S. & Figueiredo, L. M. Circadian rhythms in parasites. *PLoS Pathog.* **13**, e1006590 (2017). 29023533 10.1371/journal.ppat.1006590 PMC5638552

[35] Rijo-Ferreira, F., Pinto-Neves, D., Barbosa-Morais, N. L., Takahashi, J. S. & Figueiredo, L. M. *Trypanosoma brucei* metabolism is under circadian control. *Nat. Microbiol.* **2**, 17032 (2017). 28288095 10.1038/nmicrobiol.2017.32 PMC5398093

[36] Arca, B., Lombardo, F., Struchiner, C. J. & Ribeiro, J. M. Anopheline salivary protein genes and gene families: an evolutionary overview after the whole genome sequence of sixteen *Anopheles* species. *BMC Genomics* **18**, 153 (2017). 28193177 10.1186/s12864-017-3579-8 PMC5307786

[37] Ribeiro, J. M. & Nussenzveig, R. H. The salivary catechol oxidase/peroxidase activities of the mosquito *Anopheles albimanus*. *J. Exp. Biol.* **179**, 273–287 (1993). 8393473 10.1242/jeb.179.1.273

[38] Calvo, E., Mans, B. J., Andersen, J. F. & Ribeiro, J. M. Function and evolution of a mosquito salivary protein family. *J. Biol. Chem.* **281**, 1935–1942 (2006). 16301315 10.1074/jbc.M510359200

[39] Isawa, H., Yuda, M., Orito, Y. & Chinzei, Y. A mosquito salivary protein inhibits activation of the plasma contact system by binding to factor XII and high molecular weight kininogen. *J. Biol. Chem.* **277**, 27651–27658 (2002). 12011093 10.1074/jbc.M203505200

[40] Calvo, E., Mans, B. J., Ribeiro, J. M. & Andersen, J. F. Multifunctionality and mechanism of ligand binding in a mosquito antiinflammatory protein. *Proc. Natl Acad. Sci. USA* **106**, 3728–3733 (2009). 19234127 10.1073/pnas.0813190106 PMC2656148

[41] Das, S. et al. Transcriptomic and functional analysis of the *Anopheles gambiae* salivary gland in relation to blood feeding. *BMC Genomics* **11**, 566 (2010). 20946652 10.1186/1471-2164-11-566 PMC3091715

[42] Ghosh, A. K. et al. Malaria parasite invasion of the mosquito salivary gland requires interaction between the *Plasmodium* TRAP and the *Anopheles* saglin proteins. *PLoS Pathog.* **5**, e1000265 (2009). 19148273 10.1371/journal.ppat.1000265 PMC2613030

[43] Brennan, J. D., Kent, M., Dhar, R., Fujioka, H. & Kumar, N. *Anopheles gambiae* salivary gland proteins as putative targets for blocking transmission of malaria parasites. *Proc. Natl Acad. Sci. USA* **97**, 13859–13864 (2000). 11087838 10.1073/pnas.250472597 PMC17666

[44] Mitchell, S. N. et al. Mosquito biology. Evolution of sexual traits influencing vectorial capacity in anopheline mosquitoes. *Science* **347**, 985–988 (2015). 25722409 10.1126/science.1259435 PMC4373528

[45] Bogale, H. N. et al. Transcriptional heterogeneity and tightly regulated changes in gene expression during *Plasmodium berghei* sporozoite development. *Proc. Natl Acad. Sci. USA* **118**, e2023438118 (2021). 33653959 10.1073/pnas.2023438118 PMC7958459

[46] Gomes-Santos, C. S. et al. Transition of *Plasmodium* sporozoites into liver stage-like forms is regulated by the RNA binding protein Pumilio. *PLoS Pathog.* **7**, e1002046 (2011). 21625527 10.1371/journal.ppat.1002046 PMC3098293

[47] Ruberto, A. A. et al. Single-cell RNA sequencing reveals developmental heterogeneity among *Plasmodium berghei* sporozoites. *Sci. Rep.* **11**, 4127 (2021). 33619283 10.1038/s41598-021-82914-w PMC7900125

[48] Lindner, S. E. et al. Transcriptomics and proteomics reveal two waves of translational repression during the maturation of malaria parasite sporozoites. *Nat. Commun.* **10**, 4964 (2019). 31673027 10.1038/s41467-019-12936-6 PMC6823429

[49] Roth, A. et al. Unraveling the *Plasmodium vivax* sporozoite transcriptional journey from mosquito vector to human host. *Sci. Rep.* **8**, 12183 (2018). 30111801 10.1038/s41598-018-30713-1 PMC6093925

[50] Amino, R., Thiberge, S., Shorte, S., Frischknecht, F. & Menard, R. Quantitative imaging of *Plasmodium* sporozoites in the mammalian host. *C. R. Biol* **329**, 858–862 (2006). 17067928 10.1016/j.crvi.2006.04.003

[51] Hopp, C. S. et al. Longitudinal analysis of *Plasmodium* sporozoite motility in the dermis reveals component of blood vessel recognition. *eLife* **4**, e07789 (2015). 26271010 10.7554/eLife.07789 PMC4594146

[52] Ejigiri, I. et al. Shedding of TRAP by a rhomboid protease from the malaria sporozoite surface is essential for gliding motility and sporozoite infectivity. *PLoS Pathog.* **8**, e1002725 (2012). 22911675 10.1371/journal.ppat.1002725 PMC3406075

[53] Tavares, J. et al. Role of host cell traversal by the malaria sporozoite during liver infection. *J. Exp. Med.* **210**, 905–915 (2013). 23610126 10.1084/jem.20121130 PMC3646492

[54] Sultan, A. A. et al. TRAP is necessary for gliding motility and infectivity of plasmodium sporozoites. *Cell* **90**, 511–522 (1997). 9267031 10.1016/s0092-8674(00)80511-5

[55] Frenal, K., Dubremetz, J. F., Lebrun, M. & Soldati-Favre, D. Gliding motility powers invasion and egress in Apicomplexa. *Nat. Rev. Microbiol.* **15**, 645–660 (2017). 28867819 10.1038/nrmicro.2017.86

[56] Montagna, G. N., Matuschewski, K. & Buscaglia, C. A. Plasmodium sporozoite motility: an update. *Front. Biosci.* **17**, 726–744 (2012). 10.2741/3954 22201771

[57] Pino, P. et al. A multistage antimalarial targets the plasmepsins IX and X essential for invasion and egress. *Science* **358**, 522–528 (2017). 29074775 10.1126/science.aaf8675 PMC5730047

[58] Nasamu, A. S. et al. Plasmepsins IX and X are essential and druggable mediators of malaria parasite egress and invasion. *Science* **358**, 518–522 (2017). 29074774 10.1126/science.aan1478 PMC5928414

[59] Bhanot, P., Schauer, K., Coppens, I. & Nussenzweig, V. A surface phospholipase is involved in the migration of plasmodium sporozoites through cells. *J. Biol. Chem.* **280**, 6752–6760 (2005). 15590623 10.1074/jbc.M411465200

[60] Hawking, F., Worms, M. J. & Gammage, K. 24- and 48-hour cycles of malaria parasites in the blood; their purpose, production and control. *Trans. R. Soc. Trop. Med. Hyg.* **62**, 731–765 (1968). 4389153 10.1016/0035-9203(68)90001-1

[61] Bozdech, Z. et al. The transcriptome of the intraerythrocytic developmental cycle of *Plasmodium falciparum*. *PLoS Biol.* **1**, E5 (2003). 12929205 10.1371/journal.pbio.0000005 PMC176545

[62] Hoo, R. et al. Integrated analysis of the *Plasmodium* species transcriptome. *EBioMedicine* **7**, 255–266 (2016). 27322479 10.1016/j.ebiom.2016.04.011 PMC4909483

[63] Le Roch, K. G. et al. Discovery of gene function by expression profiling of the malaria parasite life cycle. *Science* **301**, 1503–1508 (2003). 12893887 10.1126/science.1087025

[64] Llinás, M., Bozdech, Z., Wong, E. D., Adai, A. T. & DeRisi, J. L. Comparative whole genome transcriptome analysis of three *Plasmodium falciparum* strains. *Nucleic Acids Res.* **34**, 1166–1173 (2006). 10.1093/nar/gkj517 PMC1380255 16493140

[65] Rijo-Ferreira, F. et al. The malaria parasite has an intrinsic clock. *Science* **368**, 746–753 (2020). 32409471 10.1126/science.aba2658 PMC7409452

[66] Nunes-Cabaco, H., Moita, D. & Prudencio, M. Five decades of clinical assessment of whole-sporozoite malaria vaccines. *Front. Immunol.* **13**, 977472 (2022). 36159849 10.3389/fimmu.2022.977472 PMC9493004

[67] Amino, R. et al. Host cell traversal is important for progression of the malaria parasite through the dermis to the liver. *Cell Host Microbe* **3**, 88–96 (2008). 18312843 10.1016/j.chom.2007.12.007

[68] Adepoju, P. RTS,S malaria vaccine pilots in three African countries. *Lancet* **393**, 1685 (2019). 31034365 10.1016/S0140-6736(19)30937-7

[69] Cohen, J., Nussenzweig, V., Nussenzweig, R., Vekemans, J. & Leach, A. From the circumsporozoite protein to the RTS, S/AS candidate vaccine. *Hum. Vaccin.* **6**, 90–96 (2010). 19806009 10.4161/hv.6.1.9677

[70] Frevert, U. et al. Malaria circumsporozoite protein binds to heparan sulfate proteoglycans associated with the surface membrane of hepatocytes. *J. Exp. Med.* **177**, 1287–1298 (1993). 8478608 10.1084/jem.177.5.1287 PMC2190997

[71] Coppi, A. et al. Heparan sulfate proteoglycans provide a signal to *Plasmodium* sporozoites to stop migrating and productively invade host cells. *Cell Host Microbe* **2**, 316–327 (2007). 18005753 10.1016/j.chom.2007.10.002 PMC2117360

[72] Foth, B. J. et al. Quantitative time-course profiling of parasite and host cell proteins in the human malaria parasite *Plasmodium falciparum*. *Mol. Cell. Proteom.* **10**, M110.006411 (2011). 10.1074/mcp.M110.006411 PMC3149090 21558492

[73] Bunnik, E. M. et al. Polysome profiling reveals translational control of gene expression in the human malaria parasite *Plasmodium falciparum*. *Genome Biol.* **14**, R128 (2013). 24267660 10.1186/gb-2013-14-11-r128 PMC4053746

[74] Le Roch, K. G. et al. Global analysis of transcript and protein levels across the *Plasmodium falciparum* life cycle. *Genome Res.* **14**, 2308–2318 (2004). 15520293 10.1101/gr.2523904 PMC525690

[75] Mueller, A. K., Labaied, M., Kappe, S. H. & Matuschewski, K. Genetically modified *Plasmodium* parasites as a protective experimental malaria vaccine. *Nature* **433**, 164–167 (2005). 15580261 10.1038/nature03188

[76] Mueller, A. K. et al. Plasmodium liver stage developmental arrest by depletion of a protein at the parasite–host interface. *Proc. Natl Acad. Sci. USA* **102**, 3022–3027 (2005). 15699336 10.1073/pnas.0408442102 PMC548321

[77] Lindner, S. E. et al. Total and putative surface proteomics of malaria parasite salivary gland sporozoites. *Mol. Cell. Proteom.* **12**, 1127–1143 (2013). 10.1074/mcp.M112.024505 PMC3650326 23325771

[78] Acosta-Rodríguez, V. A., Rijo-Ferreira, F., Green, C. B. & Takahashi, J. S. Importance of circadian timing for aging and longevity. *Nat. Commun.* **12**, 2862 (2021). 34001884 10.1038/s41467-021-22922-6 PMC8129076

[79] Thomsen, E. K. et al. Mosquito behavior change after distribution of bednets results in decreased protection against malaria exposure. *J. Infect. Dis.* **215**, 790–797 (2017). 28007921 10.1093/infdis/jiw615 PMC5388271

[80] Janse, C. J., Ramesar, J. & Waters, A. P. High-efficiency transfection and drug selection of genetically transformed blood stages of the rodent malaria parasite *Plasmodium berghei*. *Nat. Protoc.* **1**, 346–356 (2006). 17406255 10.1038/nprot.2006.53

[81] Kennedy, M. et al. A rapid and scalable density gradient purification method for *Plasmodium* sporozoites. *Malar. J.* **11**, 421 (2012). 23244590 10.1186/1475-2875-11-421 PMC3543293

[82] Dobin, A. et al. STAR: ultrafast universal RNA-seq aligner. *Bioinformatics* **29**, 15–21 (2013). 23104886 10.1093/bioinformatics/bts635 PMC3530905

[83] Martin, M. Cutadapt removes adapter sequences from high-throughput sequencing reads. *EMBnet* **17**, 10–12 (2011).

[84] Sreenivasamurthy, S. K. et al. Mosquito-borne diseases and omics: tissue-restricted expression and alternative splicing revealed by transcriptome profiling of *Anopheles stephensi*. *OMICS* **21**, 488–497 (2017). 28708456 10.1089/omi.2017.0073

[85] *gplots: Various R Programming Tools for Plotting Data* (CRAN, 2015).

[86] Wu, G., Anafi, R. C., Hughes, M. E., Kornacker, K. & Hogenesch, J. B. MetaCycle: an integrated R package to evaluate periodicity in large scale data. *Bioinformatics* **32**, 3351–3353 (2016). 27378304 10.1093/bioinformatics/btw405 PMC5079475

[87] Hughes, M. E., Hogenesch, J. B. & Kornacker, K. JTK_CYCLE: an efficient nonparametric algorithm for detecting rhythmic components in genome-scale data sets. *J. Biol. Rhythms* **25**, 372–380 (2010). 20876817 10.1177/0748730410379711 PMC3119870

[88] Yang, R. & Su, Z. Analyzing circadian expression data by harmonic regression based on autoregressive spectral estimation. *Bioinformatics* **26**, i168–i174 (2010). 20529902 10.1093/bioinformatics/btq189 PMC2881374

[89] Thaben, P. F. & Westermark, P. O. Detecting rhythms in time series with RAIN. *J. Biol. Rhythms* **29**, 391–400 (2014). 25326247 10.1177/0748730414553029 PMC4266694

[90] Huang, D. W., Sherman, B. T. & Lempicki, R. A. Systematic and integrative analysis of large gene lists using DAVID bioinformatics resources. *Nat. Protoc.* **4**, 44–57 (2009). 19131956 10.1038/nprot.2008.211

[91] Costa-da-Silva, A. L. Artificial membrane feeding mosquitoes in the laboratory with Glytube. *Cold Spring Harb. Protoc.* **2023**, 108013-pdb prot (2023). 36223987 10.1101/pdb.prot108013

[92] Mello-Vieira, J., Enguita, F. J., de Koning-Ward, T. F., Zuzarte-Luis, V. & Mota, M. M. *Plasmodium* translocon component EXP2 facilitates hepatocyte invasion. *Nat. Commun.* **11**, 5654 (2020). 33159090 10.1038/s41467-020-19492-4 PMC7648069

[93] Brown, S. A. et al. The period length of fibroblast circadian gene expression varies widely among human individuals. *PLoS Biol.* **3**, e338 (2005). 16167846 10.1371/journal.pbio.0030338 PMC1233413

[94] Ribeiro, J. M., Martin-Martin, I., Arca, B. & Calvo, E. A deep insight into the sialome of male and female *Aedes aegypti* mosquitoes. *PLoS ONE* **11**, e0151400 (2016). 26999592 10.1371/journal.pone.0151400 PMC4801386

