# Supplementary material for: Parasite and vector circadian clocks mediate efficient malaria transmission
Source: Nat Microbiol. 2025 Mar 31;10(4):882–96. doi: 10.1038/s41564-025-01949-1 (PMC11964930; doi:10.1038/s41564-025-01949-1)
Supplement: Supplementary file 1 — Reporting Summary [file 41564_2025_1949_MOESM1_ESM.pdf]

nature portfolio

Corresponding author(s): Inês Bento, Filipa Rijo-Ferreira

Last updated by author(s): Dec 16, 2024

Reporting Summary

Nature Portfolio wishes to improve the reproducibility of the work that we publish. This form provides structure for consistency and transparency in reporting. For further information on Nature Portfolio policies, see our Editorial Policies and the Editorial Policy Checklist.

Statistics

For all statistical analyses, confirm that the following items are present in the figure legend, table legend, main text, or Methods section.

n/a	Confirmed	
☐	☒	The exact sample size (*n*) for each experimental group/condition, given as a discrete number and unit of measurement
☐	☒	A statement on whether measurements were taken from distinct samples or whether the same sample was measured repeatedly
☐	☒	The statistical test(s) used AND whether they are one- or two-sided<br>*Only common tests should be described solely by name; describe more complex techniques in the Methods section.*
☐	☒	A description of all covariates tested
☐	☒	A description of any assumptions or corrections, such as tests of normality and adjustment for multiple comparisons
☐	☒	A full description of the statistical parameters including central tendency (e.g. means) or other basic estimates (e.g. regression coefficient) AND variation (e.g. standard deviation) or associated estimates of uncertainty (e.g. confidence intervals)
☐	☒	For null hypothesis testing, the test statistic (e.g. *F*, *t*, *r*) with confidence intervals, effect sizes, degrees of freedom and *P* value noted<br>*Give P values as exact values whenever suitable.*
☒	☐	For Bayesian analysis, information on the choice of priors and Markov chain Monte Carlo settings
☐	☒	For hierarchical and complex designs, identification of the appropriate level for tests and full reporting of outcomes
☒	☐	Estimates of effect sizes (e.g. Cohen's *d*, Pearson's *r*), indicating how they were calculated

Our web collection on statistics for biologists contains articles on many of the points above.

Software and code

Policy information about availability of computer code

Data collection

Sequencing libraries were constructed using the TruSeq RNA Sample preparation protocol (Illumina). RNA-sequencing of libraries was performed on the Hiseq2000 (Illumina) with 50 bp reads for the first experiment 0-48h LD and DD, and on the NextSeq 550 (Illumina) with 75 bp reads for the second experiment 0-72h LD and DD, according to the manufacturer's instructions by the UTSW McDermott Next Generation Sequencing Core. Read quality was assessed using the FASTQC quality control tool (http://www.bioinformatics.babraham.ac.uk/projects/fastqc). Reads were mapped with STAR 75 following Cutadapt trimming 76 to the following genomes: PlasmoDB-26_PbergheiANKA from www.PlasmoDB.org and Anopheles-stephensi-SDA-500_ AsteS1.6 from www.vectorbase.org.

For mass spec proteomics data: LC-MS/MS data acquisition. Samples were analyzed on a timsTOF SCP (Bruker Daltonics) coupled to an EvoSep One liquid chromatography system (Evosep Biosystems). Samples were prepared for injection by loading 20µg of peptides each onto Evotip Pures (Evosep Biosystems). Peptides were eluted online from the Evotips and separated by reverse-flow chromatography on a 15 cm PepSep column (75 µm internal diameter, 1.9 µm C18 beads; Bruker Daltonics) with a 10 µm zero-dead volume sprayer (Bruker Daltonics), using the Whisper 20 method (60-min gradient length, 100 nL/min flow rate) from Evosep. The column was maintained at 50°C in a Bruker column toaster.
Data was acquired in dda-PASEF mode (data-dependent acquisition with parallel accumulation serial fragmentation) with high sensitivity detection enabled. Ions were delivered to the timsTOF SCP through CaptiveSpray ionization and analyzed across a mass range of 100-1700 m/z and mobility range of 0.7-1.3 1/k0. Accumulation and ramp time were both set to 166 ms, with a 100% duty cycle. For each MS1 scan, 5 MS2 PASEF ramps were performed for a total cycle time of 1.03 ms. A polygon filter was used to exclude singly charged precursor species. Quadrupole isolation of precursors for MS2 fragmentation was set to allow a 2 m/z window for precursors under 700 m/z and a 3 m/z window for precursors above 800 m/z. Collision energy linearly increased with ion mobility, from 20eV at 0.6 1/k0 to 65eV at 1.6 1/k0. The intensity threshold for precursor repetitions was set to 500, with a target intensity of 20,000. Active exclusion of precursors was released after 0.2 min.

To record Bmal1 promoter luciferase, media was supplemented with 100 µM luciferin (VivoGlo, Promega) and luciferase activity was recorded at 30 min intervals using a CLARIOstar luminometer (BMG Labtech) and SMART control software version 6.20, where cells were kept at alternating 37°C/35°C and 5% CO2.
For parasite in mouse liver qpCR: Measurements of SYBR fluorescence were performed on BioRad CFX96 C1000 Real-Time PCR Systems (Bio-Rad Laboratories), visualized and exported from BioRad CFX96 Manager software version 2.1.

Data analysis

Downstream bioinformatic analysis after RPKM calculations was performed in RStudio 1.1.463. Hierarchical clustering and heatmaps were obtained for each dataset by reordering the timepoints according to gene expression using function heatmap.2 from the gplots package version 3.0.1.1 (Fig. S1 and S5). Both hierarchical clustering analysis and heatmaps of Spearman correlations were performed on centered log2-transformed RPKM values. Cycling of mRNA was assessed using four circadian statistical programs: MetaCycle package version 1.2.0, which implements Lomb-scargle, JTK_CYCLE, ARSER, and the RAIN package. version 1.40.0. Using the MetaCycle R package, each permutation was analyzed for cycling genes using the JTK, LS, and ARSER algorithms.

For proteomics data obtained from mass spec: The raw LC-MS/MS data was searched with Fragpipe 20.0 against a combined Anopheles stephensi SDA-500 (www.vectorbase.org) and Plasmodium berghei ANKA database (www.PlasmoDB.org), with contaminant and reverse sequences added by Fragpipe. Redundant protein sequences were consolidated into single FASTA entries. Entries were reformatted to allow Fragpipe to parse out relevant information, including Protein ID and description, organism, and gene name. Default parameters for closed, tryptic search were used, with the inclusion of deamidation (NQ) and phosphorylation (STY) as additional variable modifications. MS1 quantification was also performed, without match-between-runs and normalization across runs enabled. The MSstats.csv output generated by Fragpipe was used for downstream analyses. Features mapping to contaminant proteins were removed prior to data processing with the MSstats R package version 4.0.0. The dataProcess function was run with default parameters, which performs log2 intensity transformation, normalization by equalizing medians, run-level protein intensity summarization, and imputation using an accelerated failure time model. Significance testing and fold-change calculation between conditions was performed using the MSstats function groupComparison.

For parasite in mouse liver qpCR: Measurements of SYBR fluorescence were analyzed using the 2-ΔΔCt method.
For qPCR of infected hepa1-6 cells: mRNA expression was calculated relative to Gapdh expression using the 2-ΔΔCt method. Statistical analysis was performed using Prism 10.
For Bmal1 promoter luciferase analysis, results were visualized and exported from MARS software version 4.20.

To identify the genes that cycle at a transcriptional level, gene orthologs from Anopheles gambiae were obtained using Biomart from www.vectorbase.org. Overall, the functions of mosquito salivary cycling genes were analyzed by KEGG pathway enrichment and InterPro protein domain enrichment using DAVID version 6.8. By overlapping our dataset of cycling mRNAs with the Lindner 2013 dataset of sporozoite proteins expressed in the salivary gland 70, we identified 238 genes that cycle at the mRNA level and whose proteins are expressed during sporozoite stage in the salivary glands (Table S3).

Image analysis was performed using ImageJ version 1.54.

For manuscripts utilizing custom algorithms or software that are central to the research but not yet described in published literature, software must be made available to editors and reviewers. We strongly encourage code deposition in a community repository (e.g. GitHub). See the Nature Portfolio guidelines for submitting code & software for further information.

Data

Policy information about availability of data

All manuscripts must include a data availability statement. This statement should provide the following information, where applicable:

- Accession codes, unique identifiers, or web links for publicly available datasets
- A description of any restrictions on data availability
- For clinical datasets or third party data, please ensure that the statement adheres to our policy

All sequencing data are deposited in GEO datasets (www.ncbi.nlm.nih.gov/geo/query/xxxx). Accession codes will be available before publication.

Research involving human participants, their data, or biological material

Policy information about studies with human participants or human data. See also policy information about sex, gender (identity/presentation), and sexual orientation and race, ethnicity and racism.

Reporting on sex and gender	Human Red Blood Cell CPD Unit Sex and Gender Unspecified.
Reporting on race, ethnicity, or other socially relevant groupings	All socially relevant grouping information was not collected and not relevant for our study.
Population characteristics	This information was not collected.
Recruitment	This information was not collected. Blood was commercially obtained from BioIVT.
Ethics oversight	Approved by the Berkeley Environmental Health and Safety.

Note that full information on the approval of the study protocol must also be provided in the manuscript.

Field-specific reporting

Please select the one below that is the best fit for your research. If you are not sure, read the appropriate sections before making your selection.

☒ Life sciences ☐ Behavioural & social sciences ☐ Ecological, evolutionary & environmental sciences

For a reference copy of the document with all sections, see nature.com/documents/nr-reporting-summary-flat.pdf

Life sciences study design

All studies must disclose on these points even when the disclosure is negative.

Sample size	The sample size was statistically predetermined using this paper (https://pubmed.ncbi.nlm.nih.gov/29098954/), which was determined to be 13. We had a total of 32 samples.<br><br>For mouse liver experiments, there were 10-23 mice per group, with 4 independent experiments performed.<br>For parasite motility, an average of 40 parasites were imaged per replicate, with 3 replicates for each timepoint.<br>For mosquito salivation & saliva proteomics, 22-47 mosquitoes were used per replicate, with 3 replicates for each timepoint.<br>For hepa1-6 infection experiments, 9 replicates were performed per timepoint, with a total of 2 independent experiments.
Data exclusions	Genes that were considered too low of expression according to our cutoff of <0.5 RPKM were excluded from this data. This exclusion criteria was pre-established.
Replication	All attempts at replication were successful due to the validation of results by two independent experiments that were performed.
Randomization	All organisms were randomly assigned to experimental groups by splitting mosquitoes into cups for specific timepoints by a researcher that was not involved in the study. Mice were also split randomly into different groups.
Blinding	Investigator blinding to group allocation during data analysis was not possible due to the restrictions of working with high dimensional data.

Reporting for specific materials, systems and methods

We require information from authors about some types of materials, experimental systems and methods used in many studies. Here, indicate whether each material, system or method listed is relevant to your study. If you are not sure if a list item applies to your research, read the appropriate section before selecting a response.

Materials & experimental systems

n/a	Involved in the study	
☐	☒	Antibodies
☐	☒	Eukaryotic cell lines
☒	☐	Palaeontology and archaeology
☐	☒	Animals and other organisms
☒	☐	Clinical data
☒	☐	Dual use research of concern
☒	☐	Plants

Methods

n/a	Involved in the study	
☒	☐	ChIP-seq
☒	☐	Flow cytometry
☒	☐	MRI-based neuroimaging

Antibodies

Antibodies used	Anti–UIS4 (goat, 1:500, from Sicgene) (A121573). The following secondary antibodies: anti-GFP rabbit polyclonal antibody conjugated to Alexa Fluor 488 (Cat. No. A-21311) and donkey anti-goat Alexa Fluor 568 (Cat. No. A-11057) (all 1:1000 from Life Technologies, Invitrogen). Cell nuclei were stained with Hoechst 33342 (Cat. No. H3570) in 1:1000 (from Life Technologies, Invitrogen Invitrogen). Sporozoites and slides mounted using Fluoromount-G (SouthernBiotech, Cat. No. 0100-01). Click-iT™ EdU Cell Proliferation Kit for Imaging, Alexa Fluor™ 488 dye (Cat. No. C10337). EdU (Invitrogen) (Cat. No. A10044). Sporozoites were fixed and stained with mouse αPbCSP (clone 3D11, from MR4, kindly provided by Miguel Prudêncio).. αTRAP antibodies (kindly provided by Joana Tavares) and Hoechst (DNA dye).
Validation	Anti–UIS4 (goat, 1:500, from Sicgene) validation: https://www.antibodies.com/uis4-antibody-a121573. "UIS4 liver section: 50μM liver sections of a B6 mice infected with Plasmodium Berghei ANKA parasites (44 hours post infection), stained with Anti-UIS4 Antibody (1:500) followed by rabbit anti-goat IgG Antibody (1:400 | red) and nuclei (blue)."<br>on MR4<br>Invitrogen antibody performance guarantee, "As a leading provider of high-quality, validated antibodies, our products are used by researchers around the world. Our customers purchase with confidence, knowing that we stand behind the quality of our antibodies with the Invitrogen™ antibody performance guarantee. If an Invitrogen antibody does not perform in your experiment as described on our website or data sheet, we will replace the product at no cost to you, or if you prefer, we will provide you with a credit for future purchase. Our performance guarantee is valid for products purchased directly from Thermo Fisher Scientific or any of our authorized distributors."

"Fluoromount-G® is a water-soluble compound for slides mounted after a staining procedure having an aqueous final step. It is suitable for microscopy applications utilizing frozen and paraffin sections, whole mounts, and cells."

For the mouse αPbCSP (clone 3D11, from MR4, kindly provided by Miguel Prudêncio), please refer to the Malaria Research and Reference Reagent Resource Center (MR4). The following reagent was obtained through BEI Resources, NIAID, NIH: Monoclonal Anti-Plasmodium berghei 44-kDa Sporozoite Surface Protein, Clone 3D11 (produced in vitro), MRA-100A, contributed by Victor Nussenzweig. Please refer to the Certificate of Analysis for more information.

For validation of rabbit polyclonal anti-TRAP, please refer to https://doi.org/10.3390/ijms23105711 (Fig 7) for antibody validation.

Eukaryotic cell lines

Policy information about cell lines and Sex and Gender in Research

Cell line source(s)

Parasites used in this study: GFP expressing P. berghei ANKA (clone 259cl2, Pb-GFP); P. berghei ANKA resistance marker free GFP expressing line (clone 440cl1, obtained from the Leiden Malaria Research Group, http://www.pberghei.eu, 73 and P. berghei ANKA-GFP line expressing the thymidine kinase (TK) from Herpes simplex virus (Pb-GFP-TK). Cells were from vertebrate models.
Hepa1-6 cells were kindly provided by the Arruda lab (University of California, Berkeley). The Hepa1-6 original commercial source ATCC: https://www.atcc.org/products/crl-1830. ATCC, catalog: CRL-1830, lot 63048648
The Pb-GFP-TK parasite line was generated by double-homologous recombination.

Authentication

Cells were authenticated by identification of infected cells in mice and through confirmation of pathogenesis in mice. This was also done for mosquitoes.

Mycoplasma contamination

Mycoplasma contamination of cell lines used in this study were confirmed to be mycoplasma negative.

Commonly misidentified lines
(See ICLAC register)

None

Animals and other research organisms

Policy information about studies involving animals; ARRIVE guidelines recommended for reporting animal research, and Sex and Gender in Research

Laboratory animals

Parasite lines
Transfected merozoites were injected into the tail vein of one malea BALB/c mouse (6–8 weeks of age) and selected by the administration of Pyrimethamine in the mice drinking water (70µg/ml). Pyrimethamine resistant parasite population containing the correct genomic integration of TK/DHFR expressing cassette was cloned by limiting dilution and injection of one parasite per mouse (10 BALB/c male mice, 6–8 weeks of age).

Mosquito rearing and infections
Female Anopheles stephensi mosquitos 4-5 days old were allowed to bite two mice infected with Plasmodium berghei ANKA (GFPcon, 259cl2 for one hour)., strain, sex, and age of animals i

Mice
Male C57BL/6 mice were purchased from Charles River or Jackson Laboratories. Mice were maintained under specific pathogen-free conditions and housed at 23ºC with a 12h light/12h dark schedule in accordance with the European regulations concerning Federation for Laboratory Animal Science Associations, category B and the standards of the University of California Berkeley Institutional Care and Use Committee. C57BL/6J were purchased from Charles River or Jackson Laboratories.

Mosquito biting.
When allowed to bite on anesthetized infected mice (C57BL/6, male, 9-12 weeks of age) at two times of the day – at ZT4 (4h after lights on) and at Z16 (4h after lights off) – mosquitos had access to the mouse for 30 minutes in their respective light settings. number

Parasite load in mouse liver
Mice (C57BL/6, male, 9-12 weeks of age) were anesthetized with 90 mg/kg of ketamine and 5 mg/kg of xylazine. number

From sporozoite injection
Four experimental groups were designed, with 10-23 mice (C57BL/6, male, 6 weeks of age) per group: ZT4 matched sporozoites and mice, ZT16 matched sporozoites and mice, as well as two mismatched groups, meaning that sporozoites collected from ZT4 mosquitos were injected in mice at ZT16, and the complementary group. age

Reporting Summary and Methods
Young adult BALB/c mice 6-8 weeks of age were used for transfection. C57BL/6J adult male mice 6-12 weeks of age were used. Infected female Anopheles mosquitoes were used. C57BL/6J mice were purchased from Charles River or Jackson Laboratories.

Wild animals

Study did not involve wild animals.

Reporting on sex	Male mice were used. Female mosquitoes were used as only female mosquitoes transmit malaria.
Field-collected samples	Study did not involve field-collected samples.
Ethics oversight	In accordance with the European regulations concerning Federation for Laboratory Animal Science Associations, category B and the standards of the University of California Berkeley Institutional Care and Use Committee.

Note that full information on the approval of the study protocol must also be provided in the manuscript.

Plants

Seed stocks	*Report on the source of all seed stocks or other plant material used. If applicable, state the seed stock centre and catalogue number. If plant specimens were collected from the field, describe the collection location, date and sampling procedures.*
Novel plant genotypes	*Describe the methods by which all novel plant genotypes were produced. This includes those generated by transgenic approaches, gene editing, chemical/radiation-based mutagenesis and hybridization. For transgenic lines, describe the transformation method, the number of independent lines analyzed and the generation upon which experiments were performed. For gene-edited lines, describe the editor used, the endogenous sequence targeted for editing, the targeting guide RNA sequence (if applicable) and how the editor was applied.*
Authentication	*Describe any authentication procedures for each seed stock used or novel genotype generated. Describe any experiments used to assess the effect of a mutation and, where applicable, how potential secondary effects (e.g. second site T-DNA insertions, mosiacism, off-target gene editing) were examined.*
